# Deep phosphoproteome analysis of *Schistosoma mansoni* leads development of a kinomic array that highlights sex-biased differences in adult worm protein phosphorylation

**DOI:** 10.1371/journal.pntd.0008115

**Published:** 2020-03-23

**Authors:** Natasha L. Hirst, Jean-Christophe Nebel, Scott P. Lawton, Anthony J. Walker

**Affiliations:** 1 School of Life Sciences Pharmacy and Chemistry, Kingston University, Penrhyn Road, Kingston upon Thames, United Kingdom; 2 School of Computer Science and Mathematics, Kingston University, Penrhyn Road, Kingston upon Thames, United Kingdom; University of California Davis, UNITED STATES

## Abstract

Although helminth parasites cause enormous suffering worldwide we know little of how protein phosphorylation, one of the most important post-translational modifications used for molecular signalling, regulates their homeostasis and function. This is particularly the case for schistosomes. Herein, we report a deep phosphoproteome exploration of adult *Schistosoma mansoni*, providing one of the richest phosphoprotein resources for any parasite so far, and employ the data to build the first parasite-specific kinomic array. Complementary phosphopeptide enrichment strategies were used to detect 15,844 unique phosphopeptides mapping to 3,176 proteins. The phosphoproteins were predicted to be involved in a wide range of biological processes and phosphoprotein interactome analysis revealed 55 highly interconnected clusters including those enriched with ribosome, proteasome, phagosome, spliceosome, glycolysis, and signalling proteins. 93 distinct phosphorylation motifs were identified, with 67 providing a ‘footprint’ of protein kinase activity; CaMKII, PKA and CK1/2 were highly represented supporting their central importance to schistosome function. Within the kinome, 808 phosphorylation sites were matched to 136 protein kinases, and 68 sites within 37 activation loops were discovered. Analysis of putative protein kinase-phosphoprotein interactions revealed canonical networks but also novel interactions between signalling partners. Kinomic array analysis of male and female adult worm extracts revealed high phosphorylation of transformation:transcription domain associated protein by both sexes, and CDK and AMPK peptides by females. Moreover, eight peptides including protein phosphatase 2C gamma, Akt, Rho2 GTPase, SmTK4, and the insulin receptor were more highly phosphorylated by female extracts, highlighting their possible importance to female worm function. We envision that these findings, tools and methodology will help drive new research into the functional biology of schistosomes and other helminth parasites, and support efforts to develop new therapeutics for their control.

## Author summary

Schistosomes are formidable parasites that cause the debilitating and life-threatening disease human schistosomiasis. We need to better understand the cellular biology of these parasites to develop novel strategies for their control. Within cells, a process called protein phosphorylation controls many aspects of molecular communication or ‘signalling’ and is central to cellular function and homeostasis. Here, using complementary strategies, we have performed the first in-depth characterisation and functional annotation of protein phosphorylation events in schistosomes, providing one of the richest phosphoprotein resources for any parasite to date. Using this knowledge, we have developed a novel tool to simultaneously evaluate signalling processes in these worms and highlight sex-biased differences in adult worm protein phosphorylation. Several proteins were found to be more greatly phosphorylated by female worm extracts, suggesting their possible importance to female worm function. This work will help drive new research into the fundamental biology of schistosomes, as well as related parasites, and will support efforts to develop new drug or vaccine-based therapeutics for their control.

## Introduction

The neglected tropical disease human schistosomiasis (bilharzia) caused by *Schistosoma* blood parasites is an enormous global public health concern [1]. This parasitic disease affects almost 240 million people across 78 countries, with ~0.8 billion at risk of infection [2,3]. Despite targeted mass drug administration efforts with praziquantel the prevalence, intensity of infection and morbidity of disease are sometimes difficult to control, particularly over the long term [4]. Uniquely within the class Trematoda, schistosomes have separate sexes. The male and female adult worms reside permanently in copula within the mesenteric (*Schistosoma mansoni*/*Schistosoma japonicum*) or perivisceral (*Schistosoma haematobium*) venous plexus and produce hundreds to thousands of eggs per day [5,6]. Although the eggs are destined for expulsion from the host in excreta facilitating life cycle transmission via the snail intermediate host, many become trapped in host tissues eliciting immunopathological reactions [5]. These immunological responses lead to liver fibrosis, hepatosplenic inflammation and intestinal disease in the case of *S*. *mansoni*/*S*. *japonicum*, or obstructive and inflammatory disease in the urinary system in the case of *S*. *haematobium* [7]. The parasite (both larval and adult stages) is highly adapted to thrive in the human host; it feeds on host blood and possesses a complex tegument thought to play an important role in host immune evasion, osmoregulation, excretion and glucose uptake [8–11], and can respond to host growth factors and other signalling molecules [12–15]. Ultimately, the exquisite biology of the schistosome underpins its fecundity and longevity—surviving on average for five to 10 years [16], but possibly up to 30 years [7]—in the human host.

Reversible phosphorylation at serine (Ser, S; pS), threonine (Thr, T; pT) and tyrosine (Tyr, Y; pY) residues is one of the most important post-translational modifications for signal transduction within cells [17]. Protein kinases (PKs) and protein phosphatases catalyze the addition and removal of phosphate groups to substrate proteins, respectively, to transiently alter the targets’ properties such as enzyme activity, localization, conformation, interactions with other proteins, or to flag them for destruction. Therefore, perhaps unsurprisingly, given their role in cellular regulation, the eukaryotic protein kinases form one of the largest protein superfamilies. The kinome of *S*. *mansoni* comprises 268 protein kinases (~1.9% of the proteome) with the eight main eukaryotic groups (AGC, CK1, STE, CaMK, CMGC, RGC, TK, and TKL) represented [18–20]. A similar number (261) are reported in *S*. *haematobium* [21]. In schistosomes, several protein kinases have been found to play important roles in a wide range of processes including development and behaviour of the larval stages, and reproductive development, pairing and survival of the adults (reviewed in [20,22]). However, despite the first schistosome (*S*. *mansoni*) genome being published in 2009 [23,24], we only have limited knowledge of protein phosphorylation events in these important parasites, with two separate studies on *S*. *japonicum* identifying 127 phosphopeptides in 92 proteins and 180 phosphopeptides in 140 proteins, respectively [25,26]. Large scale (‘global’) phosphoproteome studies, such as those that have been completed for yeast (*Saccharomyces cerevisiae*) [27], human (*Homo sapiens*) [28,29], fly (*Drosophila melanogaster*) [30], and the human malaria parasite (*Plasmodium falciparum*) [31] have been vital to our understanding of the complex biology of these organisms as they provide novel insights into the extent and function of this important post-translational modification.

To obtain a comprehensive understanding of *in vivo* phosphorylation in *S*. *mansoni*, in the current study the entire adult worm proteome was digested enzymatically and phosphopeptides were enriched using immobilised metal affinity chromatography (IMAC)-Fe^3+^ (for pS/pT), and immunoprecipitation with phosphotyrosine (pY) motif antibody further followed by pS/pT motif antibody mix. The captured phosphopeptides were then analysed by liquid chromatography-mass spectrometry (LC-MS)/MS LTQ-Orbitrap Elite. In total 12,936 phosphorylation sites from 3,176 *S*. *mansoni* proteins were discovered. These data provide one of the deepest S/T/Y phosphopeptide resources for a parasite published to-date and yield novel insights into the range of functions regulated by S/T/Y phosphorylation in *S*. *mansoni*. Currently, it is difficult to analyse protein kinase signalling in parasites due to the limited tools available. Therefore, using the novel phosphoproteomic data generated for *S*. *mansoni*, we developed a peptide-based array to facilitate screening of kinase activities in this parasite. We reveal differences in peptide substrate phosphorylation by protein kinases from adult male and female schistosomes, highlighting for the first time the utility of kinomic arrays for the simultaneous screening of multiple parasite kinase activities.

## Results

### Depth and character of the detected *S*. *mansoni* phosphoproteome

**Fig 1:**
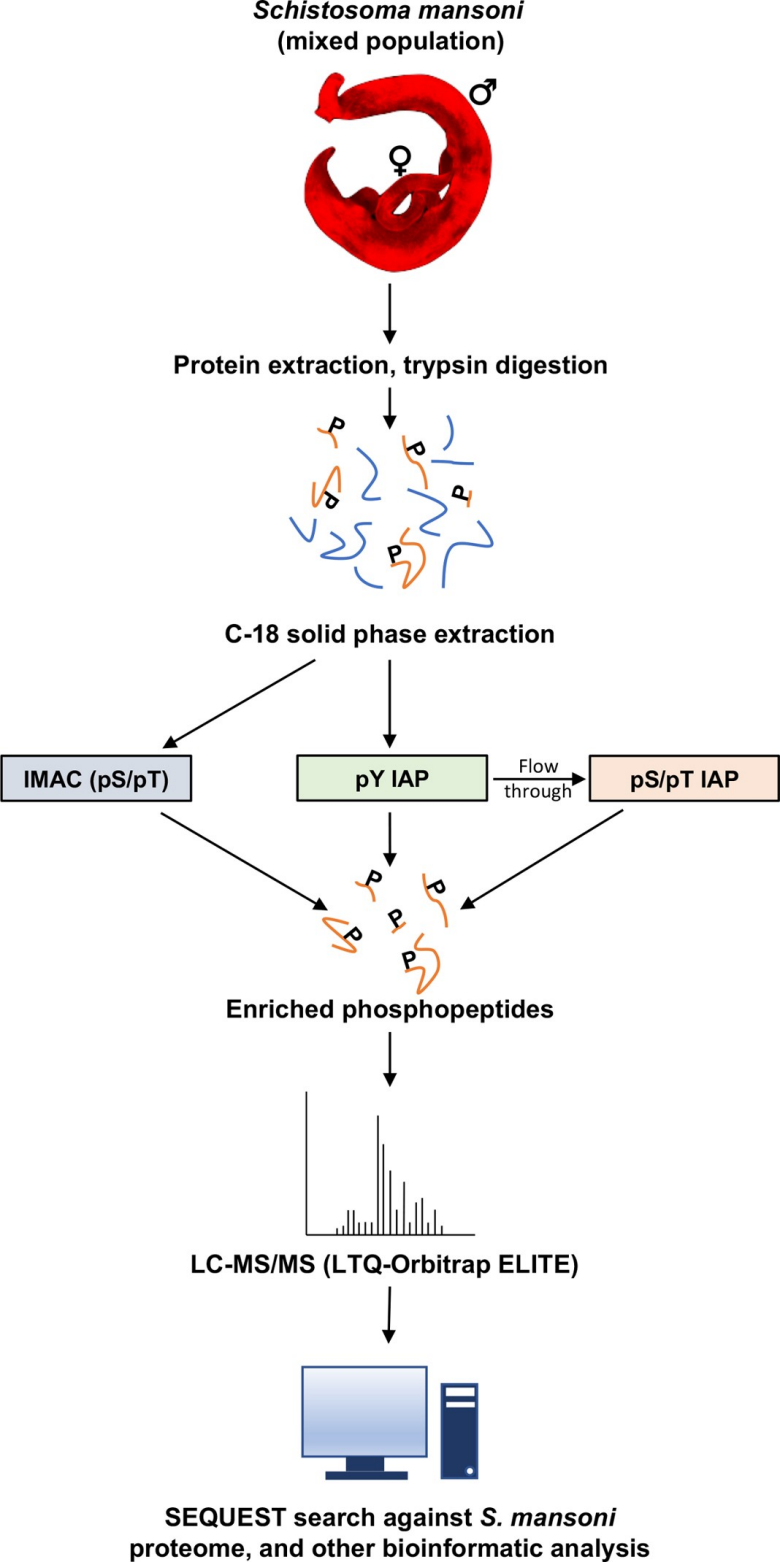
Overview of phosphoproteomic workflow employed for the large-scale detection phosphopeptides in tryptic digests of adult *S*. *mansoni*. After solid phase extraction, phosphopeptides were enriched using a combination of IMAC-Fe^3+^ and IAP with pY antibody or pS/pT (PhosphoScan) antibody mix, with the latter performed on the pY flow-through and analysed with an LTQ-Orbitrap ELITE mass spectrometer. SEQUEST and CORE were employed for phosphopeptide identification.

Proteins for phosphoproteomic analysis were extracted from a mixed population of ~400 adult male and female *S*. *mansoni*, obtained from three separate batches of mouse infections each passaged though different batches of *Biomphalaria glabrata* snails. Western blotting was then performed using a panel of three phospho-motif (phospho-Akt substrate, phospho-PKA substrate, and phospho-PKC substrate) antibodies to confirm that each of the three separate batches of extracted protein were of suitable quality for phosphoproteomic profiling (S1 Fig). Following trypsin digestion of the pooled samples, peptides were separated from non-peptide material using C18 cartridge solid-phase extraction and phosphorylated peptides isolated using a combination of IMAC-Fe^3+^ and immunoaffinity purification (IAP) with pY antibody or pS/pT antibody mix, with the latter carried out on the pY flow-through (Fig 1). This latter step of the workflow, which employed a proprietary mix of antibodies directed towards S/T phosphomotifs (PhosphoScan, Cell Signaling Technology; [32]) was done in an effort to discover additional phosphorylation sites that might have escaped detection using IMAC-Fe^3+^. Tandem mass spectra were acquired with an LTQ-Orbitrap ELITE mass spectrometer.

**Fig 2:**
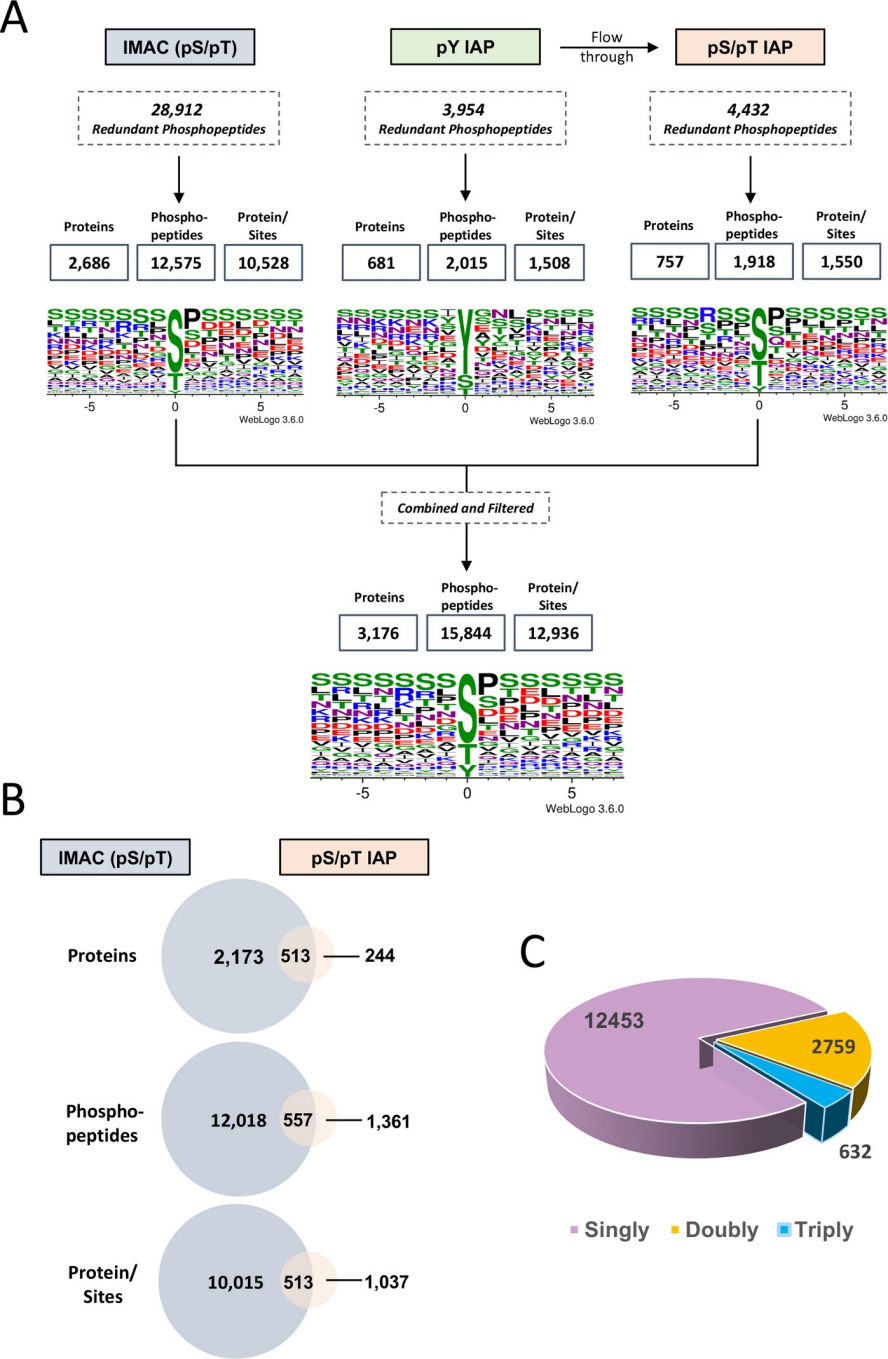
Depth and character of the detected *S*. *mansoni* phosphoproteome. (A) Individually, IMAC-Fe^3+^ enrichment resulted in the identification of considerably more phosphopeptides than antibody enrichment with pY and pS/pT IAP combined after filtering out duplicates. The three datasets were next merged and duplicates present between enrichments filtered out resulting in 15,844 unique phosphopeptides. The sequence logos depict the amino acid occurrence (-7/+7 from the phosphorylation site) amongst all phosphorylated peptides identified. (B) Data derived from IMAC-Fe^3+^ or pS/pT IAP were compared to establish the number of additional proteins, phosphopeptides, and protein/site identifications achieved with the additional pS/pT (PhosphoScan) step. (C) Unique phosphopeptides were screened to identify the numbers of peptides that were phosphorylated on either one, two, or three residues.

A total of 37,298 redundant phosphopeptides were identified using the three separate enrichment techniques (Fig 2A); search results were filtered for a 2.5% false discovery rate (FDR) and full data tables can be seen in S1–S3 Tables. The raw data were then filtered to derive protein, phosphopeptide, and protein/site data for each separate enrichment regimen (IMAC, pY IAP, and pS/pT IAP). Final inference filtering of the combined dataset revealed a total of 15,844 different phosphopeptides mapping to 3,176 *S*. *mansoni* proteins and containing 12,936 unique protein/sites (Fig 2A). These summary statistics provide an overall conservative representation of the unique phosphoproteomic data whereby a site is only counted once (even if it could appear in two or more proteins but where peptidyl coverage in the neighbourhood of the site is insufficient to confirm this). Performing pS/pT IAP after pY IAP (Fig 1) enabled approximately 10% more unique pS/pT sites to be identified than were discovered using IMAC enrichment alone (Fig 2B). Analysis of all phosphorylated peptides revealed that the majority (~78.6%) were monophosphorylated, with considerably fewer doubly or triply phosphorylated (~17.4% and ~4%, respectively) (Fig 2C).

**Fig 3:**
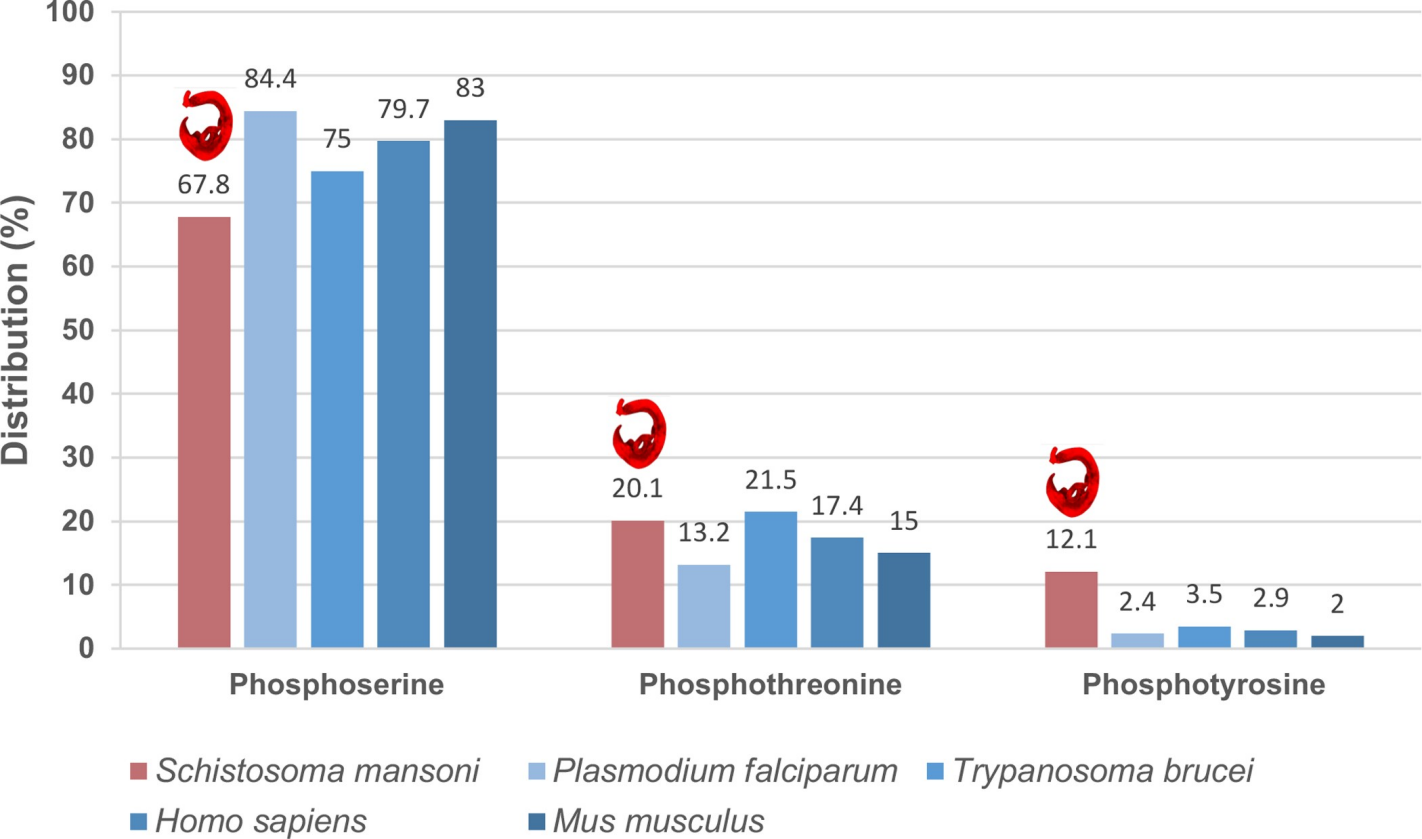
Distribution of phosphorylated serine, threonine and tyrosine residues in the *S*. *mansoni* proteome. Data were compared with that published for *Plasmodium falciparum* [31], *Trypanosoma brucei* [33], *Homo sapiens* [34], and *Mus musculus* [35].

We next investigated the amino acid distribution of *S*. *mansoni* phosphorylation sites and discovered that phosphoserine (67.8%) was the most abundant site identified followed by phosphothreonine (20.1%) and phosphotyrosine (12.1%) (Fig 3). Comparison with two other parasites (*P. falciparum* [31] and *Trypanosoma brucei* [33] and two mammals (*Homo sapiens* [34] and *Mus musculus* [35]), however, revealed that there was a somewhat greater proportion (3 to 4-fold) of phosphotyrosine identified in *S*. *mansoni* and less phosphoserine.

### Discovery of phosphorylation motifs

**Fig 4:**
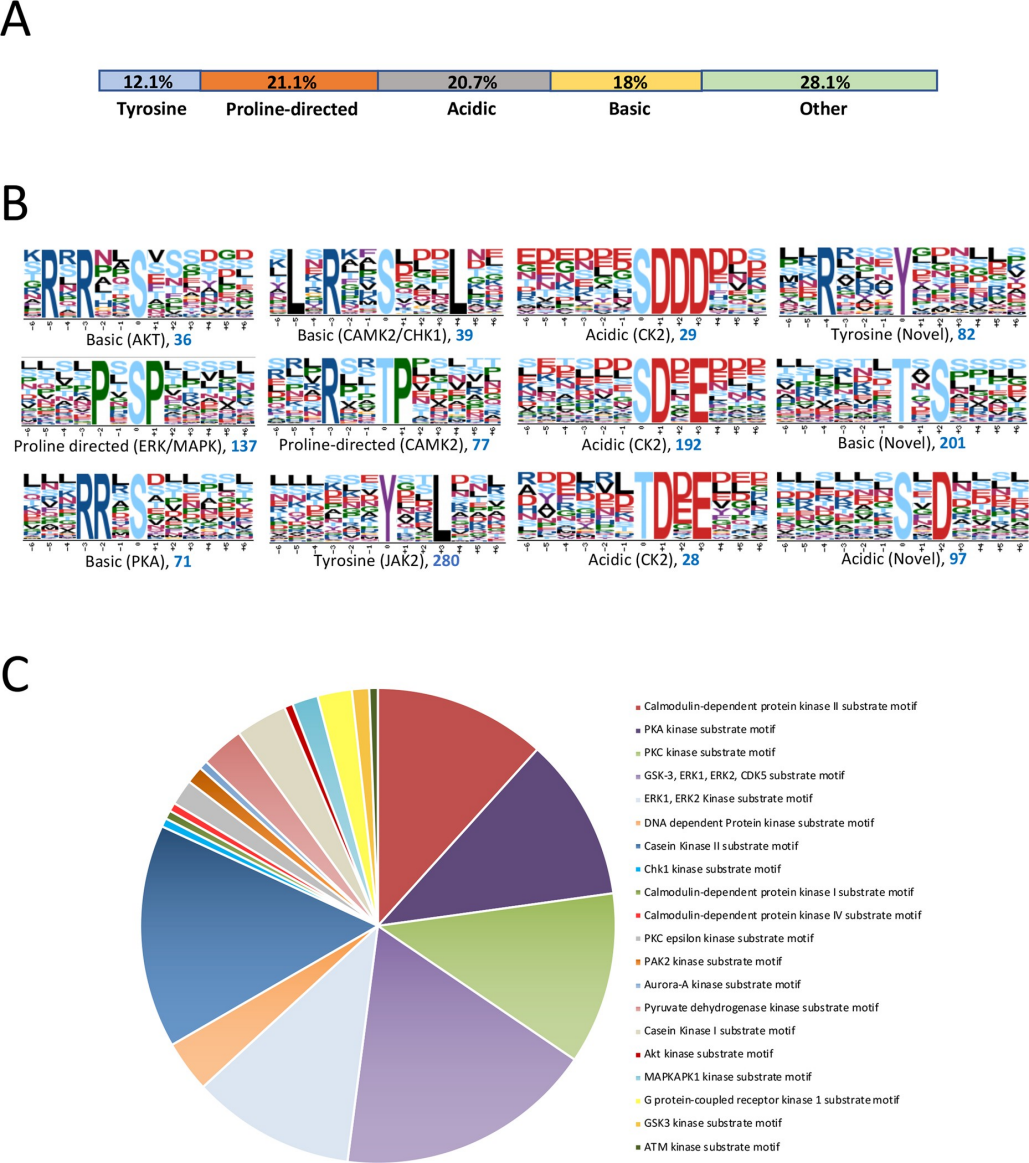
Classification of phosphorylation specific motifs and upstream protein kinases. (A) Distribution of motif classes (acidic, basic, proline-directed, tyrosine, other) based on the chemical properties of the 13 amino acid sequence window using a decision tree method [35]. (B) Significantly enriched phosphorylation motifs were generated using the Motif-X algorithm using the *S*. *mansoni* full proteome as background. Sequence logos for selected motifs are shown; occurrences are shown in blue text under each logo. The algorithm was applied to the normal amino acid set and the degenerate set (all motifs are in S4 Table). Known motifs for protein kinases (listed under each logo) were searched using the HPRD motif finder and Phosida. (C) Distribution of motifs annotated to protein kinases derived from the HPRD motif finder tool.

Next, a decision tree approach, previously employed to characterize the mouse phosphoproteome [35], was used to classify the sequences surrounding each phosphorylation site (from the -6 to +6 position) based on their chemical properties. Tyrosine motifs were the least represented (~12%), followed by basic (18%), acidic (~21%), proline-directed (~21%), and ‘other’ (~28%) (Fig 4A). Classification of phosphorylation motifs (occurrence threshold 20) was then done using the *de novo* motif searching algorithm Motif X with a 13 amino acid motif window centered on the phosphorylation site [36]. In total 4 Tyr, 65 Ser, and 24 Thr motifs (normal amino acid set) were significantly enriched at high confidence with the latter two types comprising 13 basic, 15 acidic, 18 proline-directed, and 43 ‘other’ motifs (Fig 4B and S4 Table); 60 Ser/Thr centred motifs were identified using the degenerate amino acid set. Certain motifs are common targets of specific protein kinases and thus provide a ‘footprint’ of kinase activity. Thus, using the phosphomotif discovery tools Phosida and Human Protein Reference Database (HPRD), motifs—many of which had a high number of peptides associated with them—were matched to known kinases including CAMK2, PKA, ERK/MAPK, Akt, CHK1/2, CK1/2, PKC, and GSK-3 (Fig 4B and 4C and S4 Table); CAMK2 (e.g. RxxSP, RxxTP, RxxS), PKA (e.g. RRxS, RKxS), and CK1/2 (e.g. SPxxS, TxxxS, SxxxSP) motifs were well represented in the dataset. In addition, 26 novel motifs (and 9 in the degenerate amino acid set) were identified that have not yet been described in reference species and are thus absent in Phosida and HPRD. While some of these motifs have some resemblance to known motifs for protein kinases (e.g., ……S.D.D.., CK2), others (e.g., ……pS..G…) are more unique and possibly identify a panel of unique substrates in schistosomes. Using HPRD a number of phosphorylation motifs were also discovered to possess consensus sites for protein binding. These included a Plk1 binding motif, WW domain binding motifs, and 14-3-3 domain binding motifs, that support protein-protein interactions between the phosphorylated and client proteins (S4 Table).

### Protein kinases

**Fig 5:**
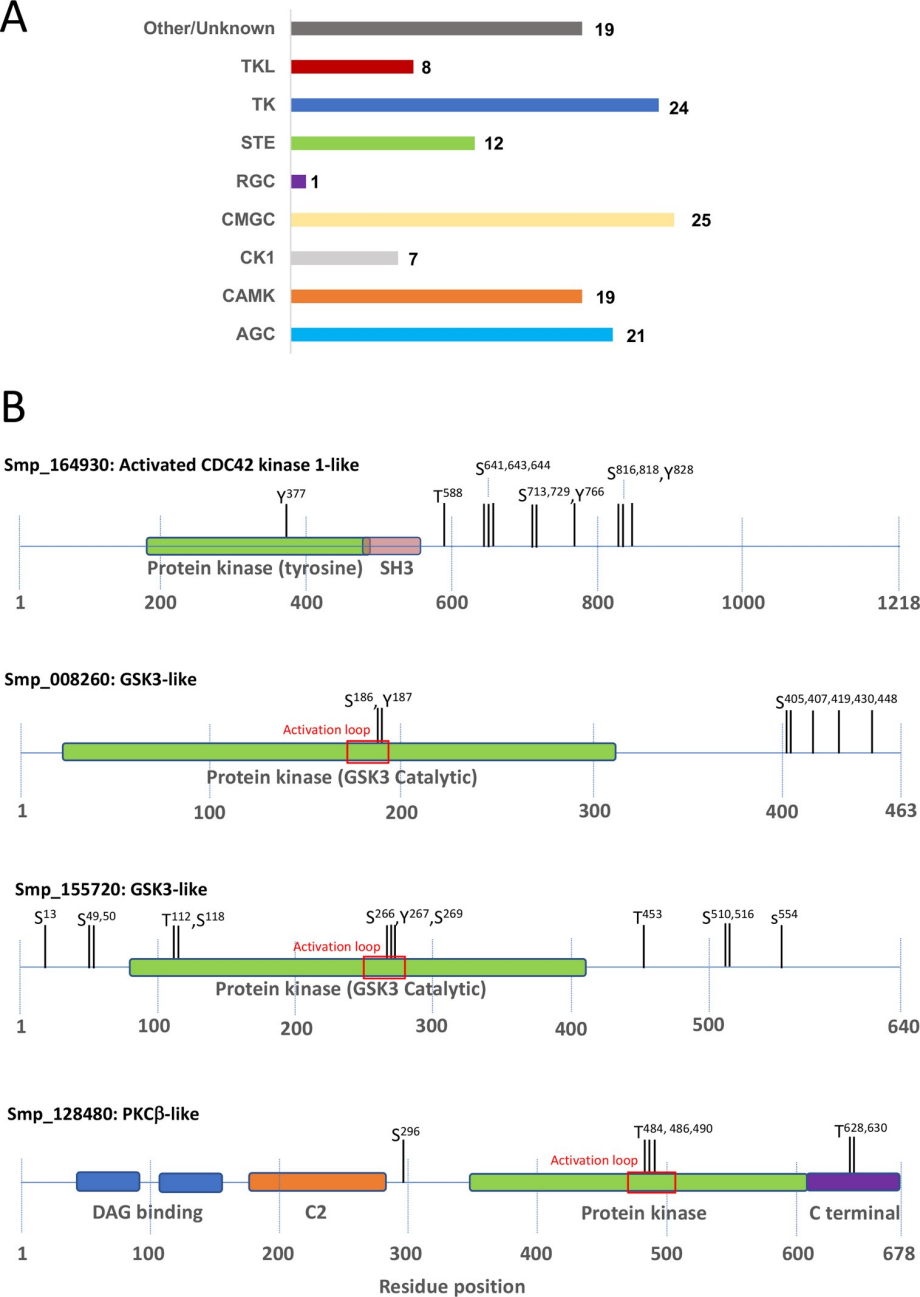
Phosphorylation of the *S*. *mansoni* kinome. (A) The number of protein kinases within each family containing identified phosphorylation sites; collation of kinases was done based upon the published kinase classification scheme for *S*. *mansoni* [18]. (B) Four examples of protein kinases displaying identified phosphorylation sites both within and outside of their protein kinase catalytic domains; phosphorylated residues discovered within the activation loops are highlighted (further details for all protein kinases are in S5 Table).

Protein phosphorylation regulates the activity and interactions of protein kinases and many become autophosphorylated upon activation [37]. Screening the phosphoproteomic dataset against the published kinome [18,20], revealed that one or more phosphorylation sites were detected in 136 of the total 268 protein kinases, with 808 phosphorylation sites (S = 531, T = 171, Y = 105) identified in total (S5 Table). Based on the classification by Andrade et al. [18], the greatest number of phosphorylated protein kinases identified (25) belonged to the CMGC followed by the TK group (24); sites were also identified in 21 AGC protein kinases (Fig 5A). Further annotation of selected protein kinases revealed that phosphorylation events were detected in the protein kinase domains, including in the activation segments and regions outside of these domains. Because many protein kinases are activated through phosphorylation of certain residues in the activation loop, which promotes an open and extended (active) conformation to facilitate substrate binding [37], we sought to identify phosphorylation events within these domains. Thus, using the conserved N-terminal activation segment ‘DFG’ motif [38] and residue annotation/conserved protein domain by InterPro/NCBI [39], respectively, to identify such loops, 68 phosphorylation sites within 37 activation loops were identified (S5 Table). To serve as examples, selected protein kinases were further annotated (Fig 5B). For example, in the β-type protein kinase C isoform (Smp_128480), which is a TDR target (Target ID: 290399; www.tdrtargets.org [40]), three close-neighbouring Thr residues were phosphorylated including the crucial Thr that is also phosphorylated in human PKCβI in the PDK1 consensus motif within the activation loop; this phosphorylation event agrees with our previous findings using ‘smart’ anti-phospho-PKC antibodies to detect activated PKC in *S*. *mansoni* [14]. Two further Thr residues were phosphorylated in the C-terminal domain, one of which (Thr^628^) corresponds to the human PKCβI autophosphorylation site and one Ser residue in the variable region. Multiple phosphorylation sites were also identified in the two *S*. *mansoni* glycogen synthase kinase 3 (GSK3)-like proteins (Smp_008260 and Smp_155720; TDR targets: 286971 and 287238, respectively). These sites include several within the activation loop of these proteins which span the sequence NVpSpYIXSR, homologous to that of humans (www.Phosphosite.org) in which the Tyr phosphorylation site regulates activity of the kinase [41]. Finally, a number of phosphorylation sites were discovered in the tyrosine kinase activated CDC42 kinase-1-like, including Tyr^377^ in the protein tyrosine kinase domain which in humans (Tyr^287^) is the primary autophosphorylation site crucial for activity (www.Phosphosite.org; [42]).

### Functional annotation of the *S*. *mansoni* phosphoproteome

**Fig 6:**
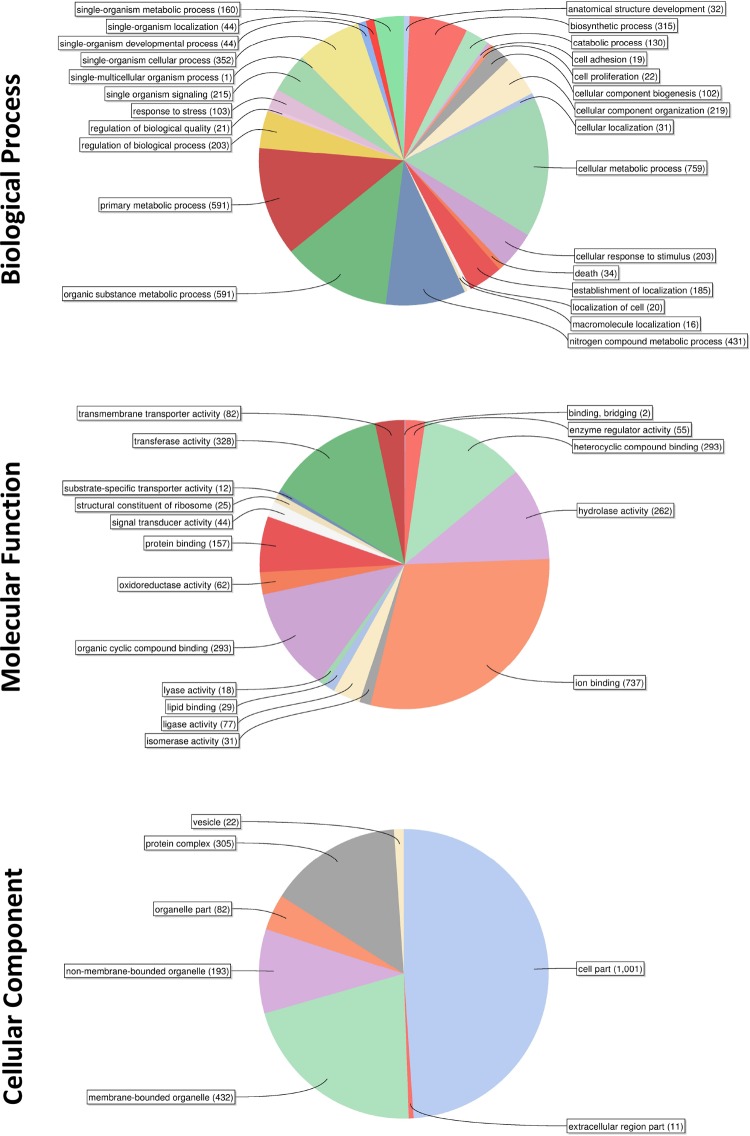
Functional annotation of the *S*. *mansoni* phosphoproteome. Gene Ontology (GO) Slim was employed using BLAST2GO to categorize identified phosphorylated proteins according to biological process, molecular function, and cellular component.

To assess the functional distribution of all identified phosphoproteins (including isoforms), functional annotation was performed by gene ontology (GO) using the annotation tool Blast2GO with GO SLIM [43]. Firstly, classification to the main functional groups (biological process, molecular function, cellular component) was performed using Ensemble Metazoa and only 6% of the phosphoproteins did not possess a GO term. The phosphoproteins were predicted to participate in a wide range of biological processes (Fig 6) with a high proportion of annotations clustering broadly with a variety of metabolic processes, biosynthetic/catabolic processes, and organizational/localization processes; other annotations included cellular responses to stress/stimulus, single organism signalling and those associated with cellular proliferation and death. In the context of molecular function (Fig 6), the predominant overarching annotation involved binding, with ion binding, bridging, heterocyclic compound binding, protein binding, organic cyclic compound binding and lipid binding all predicted; hydrolase activity and transferase activity were also highly represented. Finally, GO terms clustering under the cellular component category revealed the broad cellular distribution of *S*. *mansoni* phosphoproteins (Fig 6).

To discover phosphoprotein-associated pathways, the phosphoproteins were mapped using Kyoto Encyclopaedia of Genes and Genomes (KEGG); 51 pathways with three hits or more were highlighted by the identified phosphoproteins (S6 Table). The pathway containing the most phosphoproteins (59, including 39 enzymes) was ‘biosynthesis of antibiotics’; multiple metabolic pathways were also highlighted as were the T cell receptor, phosphatidylinositol, and mTOR signalling pathways/systems (S2 Fig).

### *S*. *mansoni* phosphoprotein interactome

**Fig 7:**
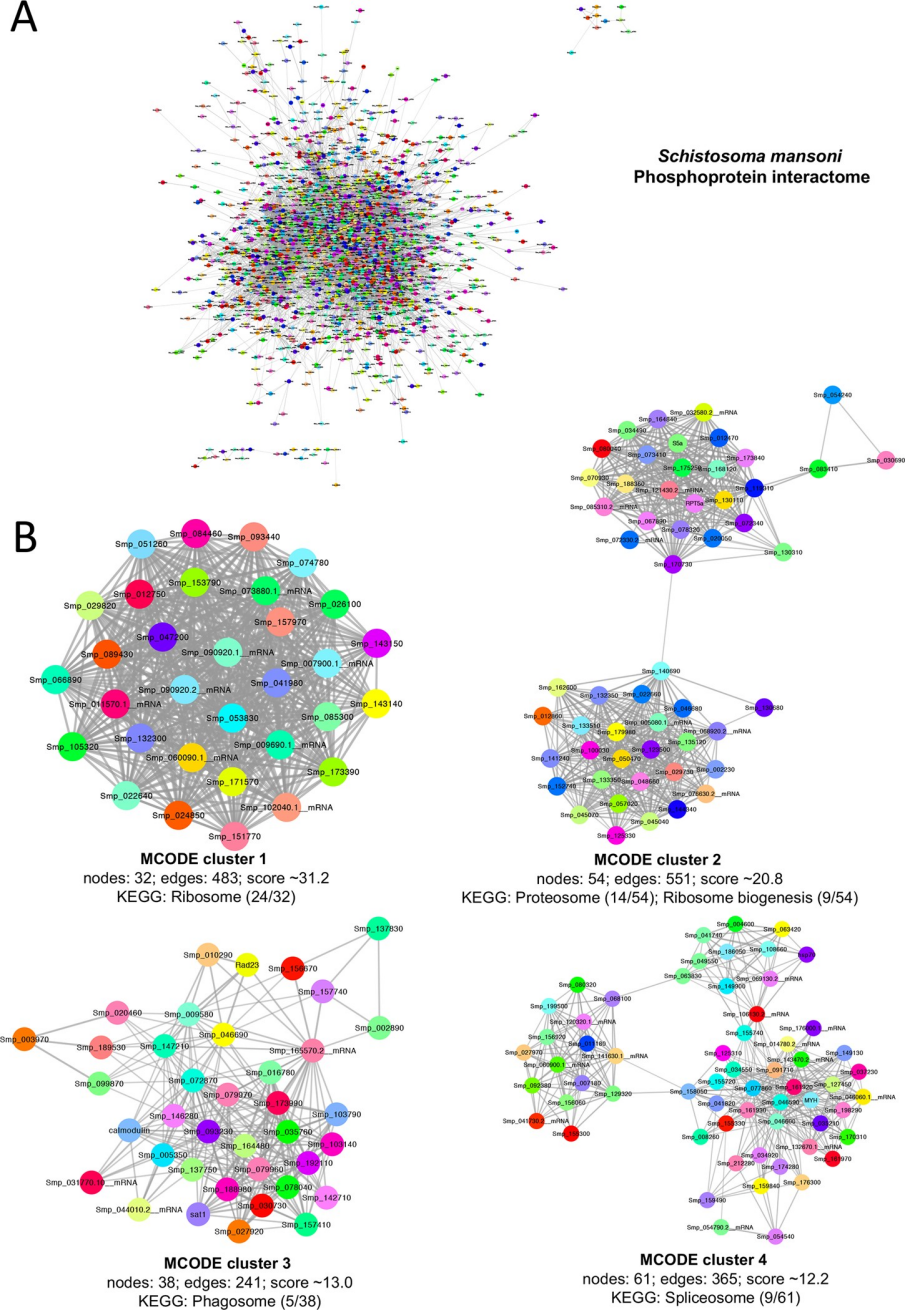
The *S*. *mansoni* phosphoprotein interaction network. (A) Protein-protein interaction data (confidence threshold 0.7) for the identified phosphorylated proteins were gathered from STRING using the “StringApp” plug-in within Cytoscape to generate the global phosphoprotein network. (B) Molecular complex detection (MCODE) using ClusterViz revealed highly interconnected sub-networks that were then functionally annotated using STRING enrichment; clusters 1–4 are shown (clusters 5–10 can be viewed in S3 Fig). The highest scoring KEGG pathway matches for each MCODE cluster are listed.

Because phosphorylation can regulate physical interactions between individual proteins (e.g. through phosphotyrosine binding domains) we next sought to discover the extent to which the phosphoproteomic data mapped onto *S*. *mansoni* protein-protein interaction data. Using Cytoscape and an app (StringApp) that extracts data from the Search Tool for the Retrieval of Interacting Genes/Proteins (STRING) database [44], complete protein-protein interaction data for *S*. *mansoni* were interrogated and a phosphoprotein interaction network was constructed by superimposing the complete phosphoproteomic data onto all putative *S*. *mansoni* protein interaction pairs at high (0.7) confidence. After removing disconnected nodes, the final phosphoprotein interactome comprised 1,586 nodes and 12,733 interactions (edges) (Fig 7A and S7 Table). Clusters of highly interconnected regions within the network were next visualized using Clusterviz which employs a molecular complex detection (MCODE) clustering algorithm. A total of 55 highly interconnected regions were detected, although all but 13 possessed MCODE scores of ≤4. The top four most connected protein complexes (clusters) are displayed in Fig 7B (clusters 4–10 are in S3 Fig, with full protein lists in S8 Table). Functional annotation revealed that MCODE cluster 1 (highest ranked) was enriched (24/32 proteins) for proteins involved in the “ribosome” KEGG pathway, whereas “proteasome” and “ribosome biogenesis” proteins were enriched in cluster 2 (Fig 7B). Proteins involved in the KEGG pathways “phagosome” and “spliceosome” were enriched in clusters 3 and 4, respectively, with other interesting pathways important to schistosome biology (e.g. “phosphoinositide signaling system”, “glycolysis”, “endocytosis”, and “SNARE interactions in vesicular transport”) over represented in clusters 5–10 (S3 Fig).

**Fig 8:**
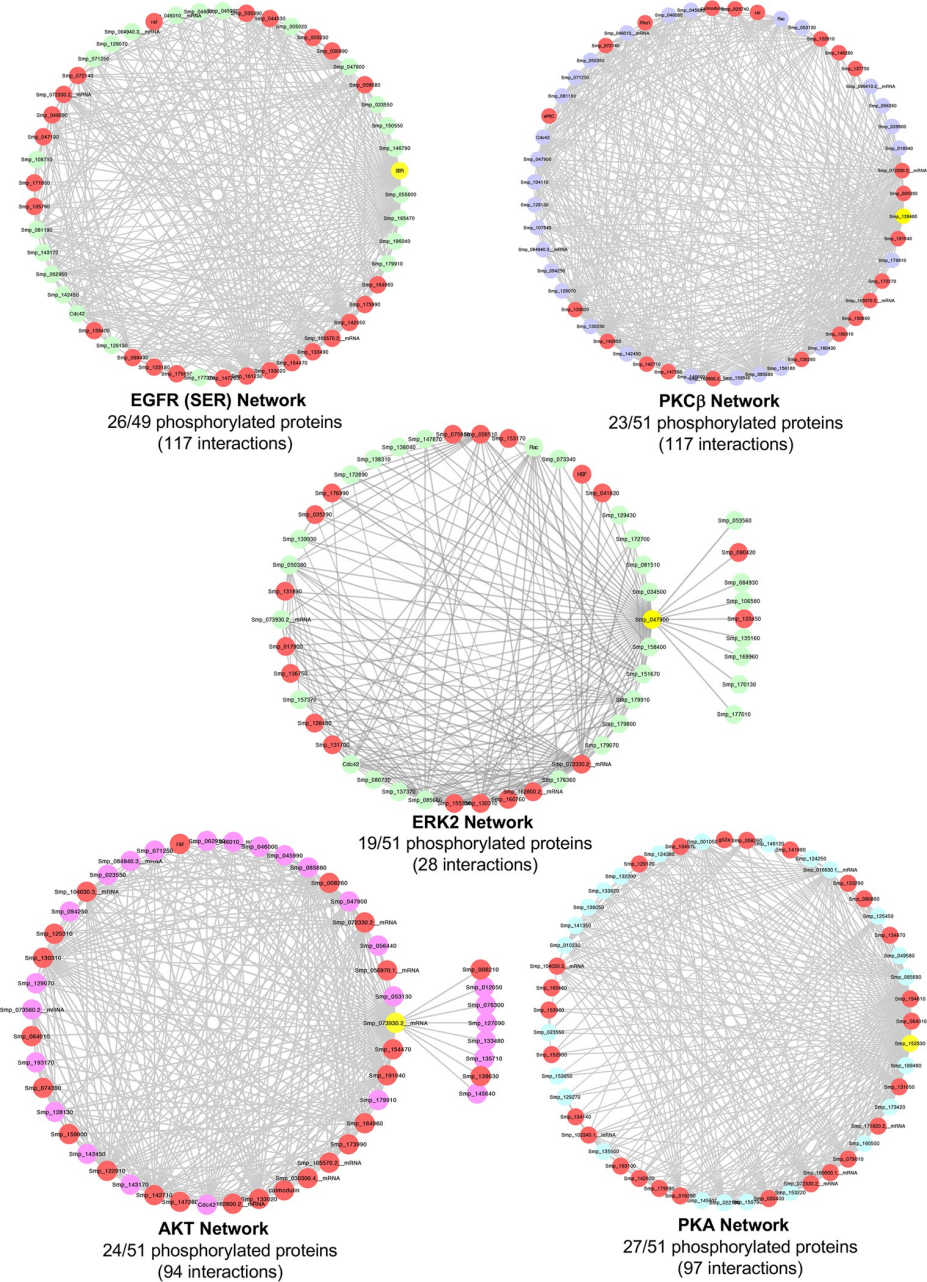
Mapping phosphorylation data onto protein-protein interaction networks. Identified phosphoproteins were mapped onto the *S*. *mansoni* STRING database of predicted protein-protein interactions for selected kinases (EGFR, PKCβ, ERK2, Akt, and PKA), using a maximum 50 interactors for each query (seed) protein and high confidence (>0.7) interaction score. Proteins (nodes) coloured red represent those phosphorylated amongst the total interaction network, with the seed node coloured yellow; note that the seed node interacts with all other proteins in the network. See also S9 Table.

The interactomes of five selected *S*. *mansoni* protein kinases were next investigated to establish the extent of phosphorylation between high-confidence interacting partners (Fig 8 and S9 Table). These kinases were chosen due to their known importance to schistosome biology: a) epidermal growth factor receptor (EGFR, SER), which binds human EGF and induces signalling in the worm [45,46]; b) PKCβ, which plays an important role in schistosome larval development and possibly regulates pairing and egg release of adults [14,47]; c) extracellular signal-regulated kinase 2 (ERK2), which together with ERK1, regulates the maintenance of the ovaries and thus egg production [48]; d) Akt, which plays an important role in glucose uptake in adult worms [15]; and e) PKA, which is important in schistosome motor activity and survival [49,50]. For each of these five networks, the identified phosphoproteins comprised between 37–53% of the predicted interactome (Fig 8). Within the EGFR/SER network, the 26 phosphoproteins included canonical members such as Grb2, Ras, Ras GTPase exchange factor Son of Sevenless (SOS), ERK, and the protein tyrosine phosphatase Shp2; other proteins included PI3K, ERK7, Rab11, and e3 ubiquitin ligase. Phosphorylated proteins that interact with PKCβ (23 proteins) included calbindins, members of the Ras and Rho GTPase families, PI4K, p38 MAPK, atypical PKC, calmodulin, and serine/threonine protein phosphatase. Phosphorylated ERK2 interactors (19 proteins) included 11 protein kinases (with 2 out of 5 cyclin dependent protein kinases being phosphorylated), 2 protein phosphatases, and 2 heat shock factors/proteins, whereas those associated with Akt (24 proteins) included Ataxia telangiectasia mutated (Atm)-related, PI3K, GSK3 and five additional protein kinases, Rab11, and GAPDH. Finally, the 27 phosphorylated proteins which clustered within the PKA network included PKA regulatory subunits, the transcription factor CREB, two camp-specific 3,5-cyclic phosphodiesterases, adenylate cyclase, Wnt, GSK3, and four protein kinases (Fig 8 and S9 Table).

### *S*. *mansoni* kinomics

**Fig 9:**
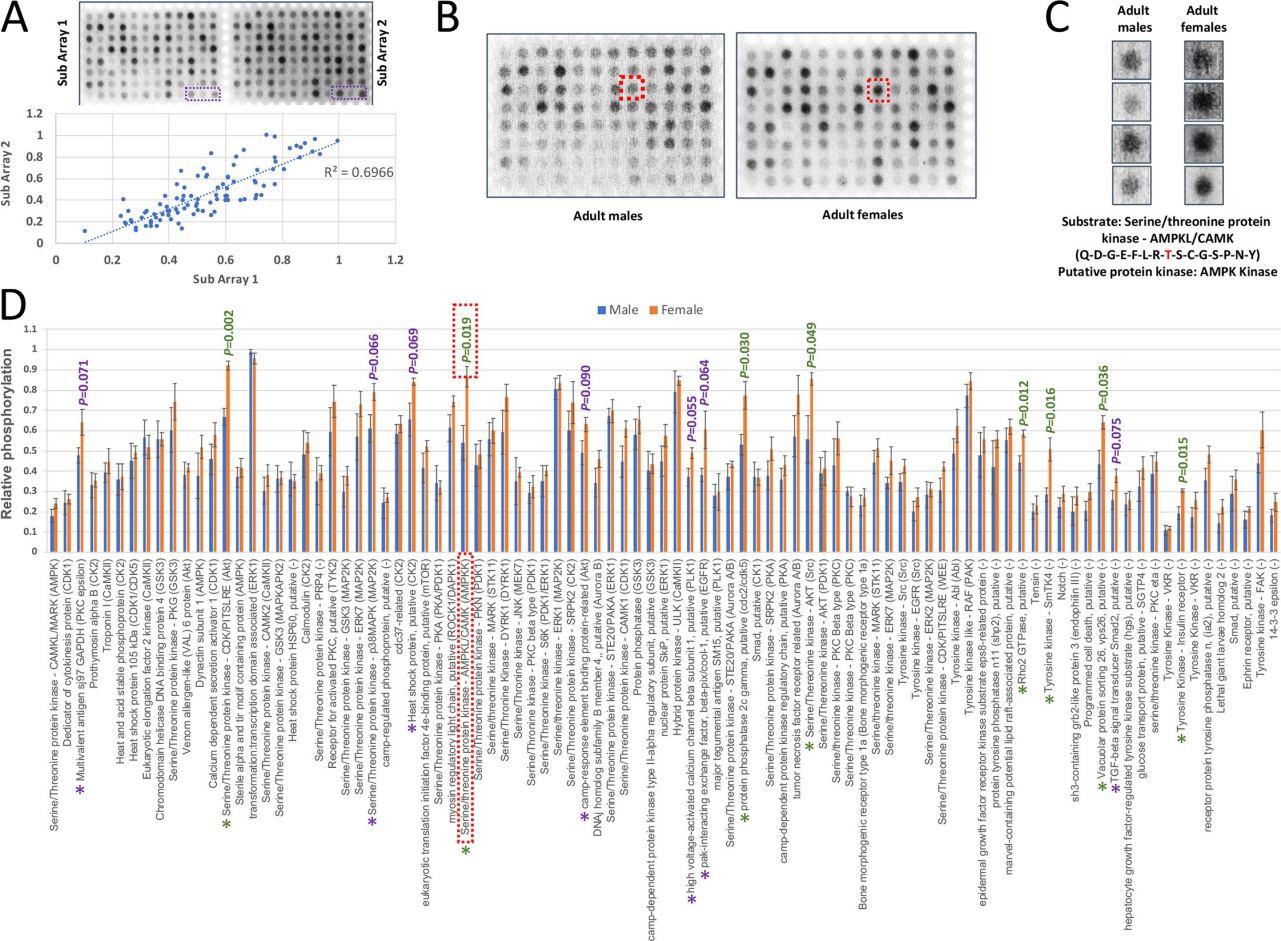
‘Chipping away’ at the *S*. *mansoni* adult worm kinome. Kinomic peptide array ‘chips’ comprising unique substrate peptides (‘spots’) for *S*. *mansoni* protein kinases were custom developed on CelluSpots slides and used to screen adult male and female protein kinase activities as detailed in Methods. (A) Representative peptide array slide incubated with female worm homogenate; 93 spots, each containing a unique peptide, were phosphorylated by worm kinases and detected using Phos-tag biotin/streptavidin HRP and ECL. Each slide comprises two sub-arrays each with identical peptides (for layout see S11 Table); the graph illustrates regression analysis of the technical replicates (normalized spot intensities) on the array. (B) Comparison of peptide sub-arrays incubated with equivalent amounts of protein (100 μg) from adult male or female worms to illustrate the different kinomic profiles of the sexes. (C) Phosphorylation of a substrate peptide sequence from the protein AMPKL/CAMK by adult male or female worm extracts; the spots shown for each sex (location highlighted in (B), red box) are from four sub-arrays; the phosphorylateable Thr residue is in red. (D) Spot intensities representing phosphorylation of each unique substrate peptide were determined using the ImageJ plugin “Protein Array Analyzer” and normalized; mean relative phosphorylation values (n = 4 ± S.E.M.) are shown and differences between mean male and female kinase activities were evaluated using Student’s t-test (green P<0.05; purple P<0.10). For each substrate peptide the putative upstream kinase (where known) is annotated within brackets. The peptide substrate in (C) (Tyrosine kinase Src) is highlighted (red box).

Using the generated phosphoproteomic data for *S*. *mansoni*, we next aimed to develop a signalling biased, *S*. *mansoni*-specific, peptide-based kinomic array to facilitate screening of kinase activities in this parasite. Such peptide arrays, developed with the knowledge that the target specificity of many protein kinases is a function of the residues in the +4 to -4 flanking positions of the phosphoacceptor site [51] (and not higher order secondary or tertiary structures), have been valuable for the high-throughput analysis of kinase activities towards defined substrates in other biological systems [52–55]. Based on substrate proteins of interest (phosphopeptides; see methods) and knowledge of their putative upstream kinases in *S*. *mansoni*, a 96-spot custom CelluSpots peptide array containing 15 mer peptides was built that was purposely biased towards cell signalling processes (S10 Table). The annotation of putative upstream kinases was based upon ScanSite and Phospho.ELM data, knowledge of comparative phosphorylation sites in signalling proteins, and our analysis of kinase/phosphorylation while investigating cell signalling in *S*. *mansoni* [14,15,22,50,56,57]. Some phosphorylation sites within the peptides (e.g. that for Abl) were autophosphorylation sites for the respective kinase. The peptide array (Fig 9A) comprised two sub-arrays each possessing identical peptides (technical replicates; substrate layout in S11 Table). The arrays were used to screen kinase-mediated substrate phosphorylation by whole adult *S*. *mansoni* (male and female) homogenates. Arrays were blocked in bovine serum albumin (BSA) and incubated with the worm extract in a humidified container at 37°C with gentle shaking for 3 h before detection of substrate peptide phosphorylation using the highly sensitive phos-tag biotin (BTL-111)-streptavidin system [58,59]. Negative control arrays lacking worm homogenate displayed minimal background signal. The correlation coefficients (r) of the two technical replicates within each analysed array ranged from 0.76 to 0.89 (linear regression), resulting in explained variance (R^2^) of 0.58 to 0.79; an example of female worm kinase-mediated phosphorylation is shown in Fig 9A (r = 0.83; R^2^ = 0.69). Arraying was typically achieved using ~10 male worm protein equivalents (comprising ~100 μg). Diluting samples by 50%, which can improve signal to noise ratios of kinomic chips [60], had no discernible benefit, instead phosphorylation levels appeared to be reduced. Peptide chips were incubated with male or female adult worm extracts to screen for peptide phosphorylation/kinase activities (Fig 9B and 9C). The peptides displaying the greatest phosphorylation overall included transformation:transcription domain associated protein in male and female worms, and the serine/threonine protein kinases CDK/PITSLRE and AMPK with putative upstream protein kinases being ERK1, Akt, and AMPKK, respectively; others included ERK1, Raf, Akt, and hybrid protein kinase ULK, with putative upstream kinases identified as MAP2K, PAK, Src, and CaMKII (Fig 9C and 9D). Overall, based upon normalized spot intensities, the female extracts tended to possess higher kinase activity towards the substrates. Furthermore, of the 93 substrate peptide substrates on the array, under the assay conditions eight were found to be differentially phosphorylated between male and female adult worm extracts, with greater phosphorylation generated by the female extracts (P<0.05). These included (fold difference and putative upstream kinase (if known) in brackets): the serine/threonine protein kinases CDK/PITSLRE (1.37-fold; Akt), AMPK (1.6-fold; AMPKK), and Akt (1.53-fold; Src); tyrosine kinases SmTK4 (1.77-fold; not known) and insulin receptor (1.57-fold; not known); protein phosphatase 2c gamma (1.47-fold; cdc2/cdk5), Rho2 GTPase (1.33-fold; not known), and vacuolar protein sorting 26 (1.47-fold; not known). At lower significance (P<0.10) a further seven peptides (multivalent antigen sj97 GAPDH, p38 MAPK, heat shock protein, camp-response element binding protein-related, high voltage-activated calcium channel beta subunit 1, pak-interacting exchange factor beta-pix/cool-1, and TGF-beta signal transducer Smad2) were differentially phosphorylated by a range of putative kinases including PKC, MAP2K, CK2, Akt, PLK1, and EGFR (Fig 9D).

## Discussion

Phosphoproteome studies have contributed significantly to developing a better understanding of the depth of phosphorylation and the function of complex cell signalling networks in a variety of organisms. Here we report the first comprehensive analysis of the phosphoproteome of the human parasite *S*. *mansoni* with 12,936 phosphorylation sites conservatively assigned to 3,176 proteins (~31% of the currently predicted 10,144 genes; 22% of 14,528 gene transcripts). While the proportion of proteins estimated to be phosphorylated within a typical proteome was previously considered to be ~30% [61], recent studies have estimated that up to ~70% of proteins may be phosphorylated on S, T, or Y residues in humans [28,62]. Thus, while to our knowledge, the current data represents the deepest S/T/Y phosphopeptide resource for any parasite published to date, many additional phosphorylated sites likely exist in *S*. *mansoni* and future advances in technology/proteomic approach will help capture these. In *S*. *mansoni*, and similar to other organisms [31,33–35], the majority of the detected phosphorylation events occurred on serine (~68%) and threonine (~20%), with fewer (12%) on tyrosine residues. However, comparatively, a greater proportion (3 to 4-fold) of phosphotyrosine was identified in *S*. *mansoni* compared to *P*. *falciparum*, *T*. *brucei*, *H*. *sapiens*, and *M*. *musculus*, with less phosphoserine. This is curious given that the number of protein tyrosine kinases (34, comprising 15 receptor tyrosine kinases and 19 cytosolic tyrosine kinases) in *S*. *mansoni* [18] as a proportion of the kinome (13.5%) is lower than that for human (90 tyrosine kinases representing ~17% of the kinome), for example [63]. Disproportionately high phosphotyrosine levels (7% of total) have also been discovered in *Dictyostelium* [64], however. Phosphorylation on tyrosine residues primarily has regulatory significance and rarely plays a structural role in proteins, is typically very transient *in vivo*, and tyrosine kinases are usually tightly negatively regulated [65]. Therefore, assuming that our approach did not technically bias the capture of p-Tyr more than in other studies, it is plausible that protein tyrosine kinases are considerably more active in *S*. *mansoni* and play a more important regulatory role than in many other eukaryotes. In this context, protein tyrosine kinases have been found to orchestrate multiple processes in schistosomes, including those central to reproductive success [20,66–71]. Although in this study we evaluated phosphorylation events in adult *S*. *mansoni*, it is important to note that the parasite transits through various life stages during its complex life cycle in human and snail hosts. Analysis of *P*. *falciparum* during intraerythrocytic development revealed stage-dependent differences in the relative proportions of p-S, p-T, and p-Y, particularly for serine phosphorylation. Thus, a global analysis of phosphorylation across all of the main *S*. *mansoni* life stages might reveal distinct stage-dependent differences.

Motif analysis of the phosphorylated peptides revealed enrichment of motifs, with 20 discovered using HPRD. These included motifs for basophilic protein kinases such as RRxS (for PKA), RxxSP and RxxTP (CAMK2), RxRxxS (Akt), and acidic protein kinases such as SDxE (CK2). Motifs for proline-directed protein kinases were also well represented including PxSP for ERK/MAPK; this motif had 137 phosphorylation sites assigned. We have previously demonstrated a role for ERK in egg laying, pairing, and movement of adult *S*. *mansoni* [14], and invasion of the host by cercariae [72]. For the tyrosine phosphorylated peptides, only one motif was discovered (YxxL) (but within 280 peptides) which HPRD revealed is a target of JAK-2 kinase. Apart from being an ancient component part of the immunoreceptor tyrosine-based activation motif (ITAM) important in phagocytosis [73] (and thus perhaps not relevant to *S*. *mansoni* which lacks specialised phagocytic cells), YxxL is also found in adaptor proteins including those that mediate insulin signalling [74,75]. Predominance of this motif may highlight novel tyrosine-dependent signalling processes that underpin the higher than usual phosphotyrosine content observed in the *S*. *mansoni* phosphoproteome. Significant populations of peptides were also identified that, based on the current motif annotations in Phosida and HPRD, lacked an associated protein kinase. Although some of the 27 ‘novel’ motifs, which included RxxxY (tyrosine), TxS (basic), and SxD (acidic), with 82, 201, and 97 peptide occurrences, respectively, somewhat resemble well-known kinase motifs, others might emerge as being unique to schistosomes highlighting schistosome-specific signalling mechanisms. Of note, and similar to here, 28 novel phosphorylated motifs were discovered in the *P*. *falciparum* proteome [31].

The majority of eukaryotic protein kinases are not constitutively activated and phosphorylation (or dephosphorylation) of certain residues either inside or outside of the activation loop governs their activation state [38,76]. Here we detected 808 phosphorylation events in ~51% (136/268 proteins) of the *S*. *mansoni* kinome, with 68 phosphorylation sites in 37 activation loops discovered. Closer inspection of selected *S*. *mansoni* protein kinases (e.g. GSK3, PKCβ, and CDC42 kinase) revealed phosphorylation site homologies with well characterised human orthologues, highlighting the evolutionary conservation of kinase regulation between the parasite and host. ‘Smart’ anti-phospho antibodies, which detect only the phosphorylated (activated) form of a particular protein kinase, are valuable tools to help characterise kinase-mediated signalling in cells/organisms. We have previously employed such antibodies to functionally study protein kinase (e.g. PKC [14,47], MAPK [14,57,72], Akt [15], PKA [13,50]) signalling in *S*. *mansoni* and the phosphorylation site data presented here offers further opportunity to either validate additional existing anti-phospho antibodies/develop new antibodies to facilitate the study of protein kinase signalling in schistosomes in various contexts (developmental, host-parasite interplay etc). Protein kinases also represent excellent drug targets and a growing number of protein kinase inhibitors have been approved for use in humans [77]; thus, protein kinases of schistosomes are being considered as possible therapeutic targets including through drug repurposing [5,20,21]. Given that phosphorylation has the potential to affect drug-target binding efficacy, particularly when within 12 Å of the binding site [78], the phosphorylation site data reported here could support the future development of drugs that target such kinases.

Schistosomes are acoelomate but possess tissues that form rudimentary ‘organ systems’ such as nerve, muscle, gut, nephridia and reproductive, to support distinct physiological processes such as excretion, feeding, locomotion and reproduction. Our phosphoproteome data show that phosphorylation occurs in a wide range of biological processes to support such physiology with a high proportion of annotations clustering with multiple metabolic, biosynthetic, and catabolic processes; other processes included signalling, cellular responses to stress/stimulus, cellular proliferation and death. Collectively, these annotations reflect the adult worm’s parasitic habit, its responses to the environment, and its enormous reproductive capacity [5] that is supported by nutrients (e.g. glucose [79]) and other essential components (e.g. lipids [23,80]—schistosomes and other platyhelminths cannot *de novo* synthesise sterols or free fatty acids) derived from the host blood. Cluster analysis using Clusterviz and MCODE revealed that the ten most interconnected complexes of phosphorylated proteins were enriched, for example, in ribosome, proteasome, phagosome, spliceosome, glycolysis, endocytosis, DNA replication, and vesicle transport processes, and phosphorylated proteins involved in glycerophospholipid and inositol phosphate metabolism. These clusters highlight the importance of protein phosphorylation to transcription/post-transcriptional control in schistosomes, degradative pathways and lipid modification/turnover. Interestingly, three phosphorylated proteins identified in Cluster 10 are involved in SNARE interactions during vesicular transport and thus might play an important part in the successful delivery of vesicles to the schistosome surface to replenish/modify the host-interactive surface layer of the parasite which turns over *in vivo*. In this context, the SNARE family member vesicle associated membrane protein 2 (VAMP2) was recently found to play a likely role in tegument maintenance, glucose uptake and egg production in *S*. *japonicum* [81]. Moreover, because the contents of schistosome-derived extracellular vesicles [82,83] have recently been found to modulate host immune cells to support parasite survival [84,85], developing an understanding of vesicle trafficking mechanisms in these worms is a priority.

The five protein kinases selected for interactome phosphorylation analysis were all TDR targets: Smp_093930 (EGFR; SER), Smp_128480 (PKCβ), Smp_047900 (ERK2), Smp_073930.2 (Akt), Smp_152330 (PKA), with associated Target IDs 282997, 290399, 283994, 324234, and 286928, respectively. These protein kinases were also chosen due to their recognised importance to schistosome biology, particularly in relation to host-parasite interactions [13,45,46], reproduction and development [14,47,48], glucose uptake [15], and motor activity and survival [49,50]. Analysis of the five networks revealed a high proportion of phosphorylated partners within each interactome. While many of the predicted interactions, such as those between EGFR and Grb2, Ras, SOS and ERK, and between PKA, PKA regulatory subunits, adenylate cyclase and CREB can be considered canonical, others are less so, offering valuable, novel, insights into the kinase regulated systems biology of *S*. *mansoni*. For example, putative routes of cross-talk between pathways are highlighted as seen between PKCβ and p38 MAPK and between Akt and Rab11. Recently, we hypothesised that Rab-GTPase family members might be activated in response to Akt stimulation to drive the delivery of the facilitated glucose transporter SGTP4 to the schistosome surface [15]. Rab11 proteins have emerged as master regulators of the cell surface expression of receptors and adhesions proteins [86] and, given the importance of the tegument in schistosome survival and host-parasite interactions, studies focusing on the regulatory mechanisms driving the activation of such Rabs are timely. Although the interactomes described here are largely derived from protein-protein binding/interaction predictions or from transferring associations/interactions between organisms (‘interlog’ transfer) [44,87], the high confidence phosphoprotein interactions discovered provide a rational framework for developing hypotheses and designing experiments to test the functional relevance of signalling processes within the parasite. Moreover, such analyses serve to highlight the importance of central players or ‘nodes’ that might be targeted by drugs or vaccines in future strategies to kill schistosomes.

Peptide kinomic arrays [88,89], developed to multiplex protein kinase activities/phosphorylation events, can yield valuable insight into the cellular regulation of organism function. Drawing upon the phosphoproteome data, peptides were selected to build an *S*. *mansoni*-specific signalling-biased array incorporating protein targets of interest. We aimed to annotate as many peptides as possible with putative upstream protein kinases, achieving annotation for 70 peptides (S10 Table); while 23 lack such annotation they were included as they represent interesting targets in terms of schistosome biology, examples being: 1) The glucose transporter SGTP4, which was recently found to be regulated by Akt [15]. 2) Smad which is involved in TGFβ-mediated signalling within the parasite [90,91]. 3) The insulin receptor 1 (IR1), which can be activated by host insulin and is important to schistosome survival [92–94]. And 4) two Venus kinase receptors (VKRs), which play a role in schistosome reproduction [70]. Under the conditions of the assay, kinomic screening of adult *S*. *mansoni* male and female homogenates revealed that eight (out of 93) peptides were differentially phosphorylated (P<0.05), with greater phosphorylation mediated by the female worm extracts in every case. These peptides (with putative upstream kinases in brackets where known) were from CDK/PITSLRE (Akt), AMPK (AMPKK), Akt (Src); SmTK4 (not known), insulin receptor (not known); protein phosphatase 2c gamma (cdc2/cdk5), Rho2 GTPase (not known), and vacuolar protein sorting 26 (not known). Seven additional peptides—multivalent antigen sj97 GAPDH, p38 MAPK, heat shock protein, camp-response element binding protein-related, high voltage-activated calcium channel beta subunit 1, pak-interacting exchange factor beta-pix/cool-1, and Smad2 were found to be differentially phosphorylated at a lower significance level (P≤0.10) and should therefore also be considered as being worthy of future investigation.

Research, particularly during the past decade, provides insights into some of these differentially phosphorylated proteins/putative upstream protein kinases in adult schistosomes. For example: 1) AMPK expression is down-regulated in *S*. *mansoni* isolated from immunodeficient mice suggesting that modulation of the worms’ energy metabolism may contribute to reduced growth and reproductive fitness in such hosts [95]. 2) Akt, a putative upstream kinase for CDK/PITSLRE and VAL 6 (a possible vaccine target) is highly active in the adult schistosome tegument and plays an important regulatory role in glucose uptake *via* SGTP4 [15]; inhibition of Akt also attenuates pairing and egg laying of adult *S*. *mansoni* [96]. 3) Insulin is able to activate Akt in the schistosome tegument [15] most likely *via* the insulin receptors [92], which are themselves vaccine targets [93]. 4) The Src-preferential inhibitor herbimycin A reduces mitosis and egg production by female *S*. *mansoni* [66,67]. 5) In *S*. *mansoni*, the Syk kinase, SmTK4, plays a crucial role in gametogenesis [68,96]. 6) Knockdown of p38 MAPK by RNAi caused tegmental aberrations, underdeveloped ovaries and reduced egg output by *S*. *mansoni* following development in mice [97]. The higher phosphorylation of substrates/activity of upstream protein kinases observed by female worm extracts, when compared with males, might reflect the signalling processes that underpin their reproductive biology, particularly as much of the female tissue is devoted to egg production with an egg being produced approximately every 4 min (based on 350 eggs produced/day [6]). Thus, we recommend the above targets are investigated further for the development of novel anti-schistosome therapies.

The novel peptide kinomic array developed here has enabled for the first time the simultaneous multiplexing of kinase-mediated substrate phosphorylation events in *S*. *mansoni*. The array can be further developed to incorporate a larger number of peptide substrates and/or can be employed with additional life stages of the parasite to investigate important questions relating to schistosome developmental biology, host-parasite interactions, sexual biology etc. *S*. *mansoni* is one of the three major schistosome species infecting humans. Given the conservation of protein kinases during evolution [98], the close relatedness of the *Schistosoma*, and the fact that the majority of protein kinase-substrate interactions should be well conserved between closely-related species [99], the phosphoproteome data generated are also of high value to researchers working on *S*. *haematobium* and *S*. *japonicum*. Moreover, we envision that the kinomic array could be directly applied to these species without modification, enabling fascinating studies into the comparative biology of signalling in schistosomes to be undertaken, particularly in the context of development of kinase inhibitors that target all major human-infective species of schistosome.

In this research we have utilised *S*. *mansoni* to provide a comprehensive analysis of the phosphoproteome of adult schistosomes, pathogens of considerable significance to human health. The dataset, which arguably represents the deepest phosphoproteomic resource obtained for any human parasite and platyhelminth to date, yields valuable and much-needed insights into the regulatory biology of *S*. *mansoni*, the best studied of the human-infective *Schistosoma* parasites. The dataset should also facilitate and support phosphoproteomic studies on related helminths. In parallel, the extensive phosphorylation site data has enabled development of a novel kinomic array for screening protein phosphorylation and kinase activity in *S*. *mansoni*; this peptide-based array is also the first kinomic array ever developed for a parasite. We demonstrate differential phosphorylation of target proteins in adult male and female *S*. *mansoni* by upstream protein kinases and propose that these be investigated further to lead the development of new anti-schistosome therapeutics.

## Materials and methods

### Ethics statement

Adult *S*. *mansoni* (Puerto Rican strain hosted by *Mus Musculus*) were obtained from BIOGLAB (c/o Prof Mike Doenhoff, University of Nottingham, UK). Laboratory animal use was regulated by the UK Animals (Scientific Procedures) Act 1986 and complied with all requirements therein. The University of Nottingham Ethical Review Committee approved mouse experiments done under Home Office license 40/3595.

### Sample preparation

The mixed male and female adult worm population (~400 worms total, obtained from three separate infections of mice) comprising couples and singles was homogenized at 4°C in groups of 10 worms each in 100 μl of urea lysis buffer (20 mM HEPES (pH 8.0), 9 M urea (Sequanal grade; Thermo Scientific), 1 mM Β-glycerophosphate) containing phosphatase inhibitors (1 mM sodium orthovanadate, 1 mM sodium pyrophosphate), using a motorized microfuge pestle (Kimble-Chase). Samples were then centrifuged at 20,000 x *g* for 10 min at 4°C, and the soluble (supernatant) fraction recovered and pooled. Aliquots of homogenate from each batch were removed for protein estimation (20 μl; Bradford assay) and for Western blot analysis (30 μl). Samples were then stored at -80°C. The pooled batches of worms yielded ~14 mg protein (~2.28 mg/ml), sufficient for a single global phosphoproteomic analysis with technical replication.

### Phosphoproteomic analysis

Phosphoproteomic analysis was done under contract by Cell Signaling Technology (CST); antibody-based IAP (via PTMScan Discovery—PhosphoScan) and IMAC were performed to isolate phosphoproteins [32,100,101] (Fig 1).

A total of 0.5 mg protein was used for enrichment by IMAC, with 13 mg used for pY IAP (and the flow-through used for pS/pT IAP). Prepared supernatants were reduced with 4.5 mM dithiothreitol for 30 min at 55°C followed by alkylation with 10 mM iodoacetamide for 15 min in the dark at room temperature. After four-fold dilution in 20 mM HEPES (pH 8.0) samples were digested overnight at room temperature with 10 μg/ml trypsin-TPCK (Worthington). Resultant peptides were acidified with trifluoroacetic acid (TFA; 1%) and desalted by solid-phase extraction with Sep-Pak C18 cartridges (Waters); eluted peptides were next dried under vacuum and stored at -80°C.

Either pY or pS/pT mix (Fig 1; PhosphoScan) were used for immunoprecipitations. Saturating amounts of the antibodies were bound to 40 μl packed protein A agarose beads (Roche) overnight at 4°C. Total peptides were resuspended in MOPS IAP buffer (50 mM MOPS, pH7.2, 10 mM KH_2_PO_4_, 50 mM NaCl) and centrifuged (5 min, 10,000 x *g*). Phosphopeptides were then enriched by mixing the sample with the antibody bead slurries (2 h at 4°C) and pulse centrifuging (30 s, 2,000 x *g*, 4°C) and washing (two washes with 1 ml MOPS IAP buffer and four washes with 1 ml MQ-H_2_O (Burdick and Jackson)). Isolated peptides were then sequentially eluted with TFA (0.15%; 65 μl then 55 μl, 10 min each) at room temperature, and desalted/concentrated over spin tips packed with Empore C18 (Sigma) and eluted with 40% acetonitrile in 0.1% Trifluoracetic acid (TFA). Eluted peptides were dried under vacuum.

For enrichment of phosphopeptides (pS/pT) by IMAC (Fig 1) [102], nickel-agarose beads (Invitrogen) were first ethylenediaminetetraacetic acid (EDTA)-treated to displace nickel, washed thrice with H_2_O, loaded with FeCl_2_ (aqueous) for 30 min, and rewashed. To enrich phosphopeptides, 10 μl Fe^3+^-agarose slurry was mixed with peptide in 1 ml 0.1% TFA/80% acetonitrile for 30 min at room temperature. The beads were then washed thrice with 0.1% TFA/80% MeCN to remove unbound peptides and bound peptides were eluted twice sequentially using 50 μl of 2.5% ammonia/50% acetonitrile for 5 min each and dried under vacuum. The samples were then resuspended in TFA (100 μl 0.15% + 2 μl 20%), desalted/concentrated and dried as previously. Finally, the peptides were resuspended in formic acid (0.125%) for LC-MS/MS.

The enriched phosphopeptides from the pooled worms were analyzed on an LTQ-Orbitrap ELITE mass spectrometer running XCallibur 2.0.7 SP1 (Thermo Scientific), with duplicate (technical) analytical injections run non-sequentially for each enriched sample run to increase the number of identifications. The peptides were loaded directly onto a PicoFrit capillary column (10 cm x 75 μm; New Objective) packed with Magic C_18_ AQ reverse-phase resin. The peptides were eluted with a 150 min linear gradient of acetonitrile in 0.125 formic acid delivered at constant flow rate of 280 nl/min. Tandem mass spectra were collected in a data-dependent manner using a top 20 MS/MS method, with the following parameters: normalized collision energy, 35%; activation Q, 0.25; and activation time, 20 ms; repeat duration, 35 s; dynamic repeat count, 1. Real time recalibration of mass error was performed using lock mass with a singly charged polysiloxane ion (m/z = 371.101237).

The MS/MS spectra were evaluated using SEQUEST [103], and CORE (Harvard University) [104] as previously detailed for PTMScan [32,101]. Briefly, searches were done against the *S*. *mansoni* genomic database (Release 5, December 2013; EnsemblMetazoa; www.metazoa.ensembl.org) with a mass accuracy of +/- 5 ppm for precursor ions and 1 Da for product ions. Enzymes specificity was limited to trypsin, cysteine carboxamidomethylation was specified a fixed modification; oxidation of methionine and the appropriate PTM were permitted as variable modifications. Reverse decoy databases were included to estimate FDR, and initially filtered at 2.5% FDR using ProteinSieve within CORE.

### Motif analysis

The chemical properties of identified phosphorylation sites were classified as acidic, basic, proline-directed, tyrosine, or other using the following published [35] decision tree approach: (1) the 6 neighbouring amino acids before and after the phosphorylation site were obtained; (2) if pY present at position (0) then = “tyrosine”; (3) if P present at position +1 then = “proline-directed”; (4) if positions +1 to +6 contain at least 2 D and E residues then = “acidic”; (5) if K or R at position -3 then = “basic”; (6) if D or E present at position +1, +2, or +3 then = “acidic”; (7) if between -6 and -1 at least 3 K or R residues exist then = “basic”; (8) remaining peptides = “other”.

To identify overrepresented phosphorylation motifs within the data set, the Motif-X algorithm was employed using default parameters (motif window = 13 amino acids; *p* value threshold for significance = 1 x 10^−6^ for S, T, or Y residues; occurrence threshold = 20) against a background of all *S*. *mansoni* proteins (Proteome ID:UP000008854) [24]. Both normal and degenerate amino acid sets were used, enabling conservative amino acid substitutions, within the motif window according to the following: A = AG, D = DE, F = FY, K = KR, I = ILVM, Q = QN, S = ST, C = C, H = H, P = P, W = W [31]. Phosida (www.phosida.de) motif matcher and the HPRD database (www.hprd.org) phosphomotif finder were next interrogated with the generated motifs to enable matching to known kinase motifs. Finally, the non-redundant raw phosphorylated peptide lists were submitted to Scansite (www.scansite4.mit.eduto) at high stringency (searching against mammalian kinases/domains) to further identify motifs recognized by upstream kinases.

### Phosphoproteome functional annotation and analysis of protein kinases

Phosphoprotein identifiers (Smp numbers) were imported into Blast2GO [43] and a cloudburst (BLASTp) conducted to map proteins and derive functional GO Slim annotations for phosphoproteins categorized into “biological process”, “cellular component” and “molecular function”. KEGG analysis was also performed within the Blast2GO platform.

Using the *S*. *mansoni* kinome [18] as a knowledgebase, protein kinases possessing identified phosphorylation sites were tabulated. Identification of the predicted activation loop for each protein kinase was achieved by manually searching for the conserved DFG motif [38] within each sequence and/or extracting the relevant sequence from the conserved protein domain tool within NCBI BLASTp [39] and InterPro. Phosphorylation site(s) within each loop were next manually annotated with reference to each identified phosphopeptide.

### Network analysis of phosphoprotein interactions

The *S*. *mansoni* phosphoproteome interaction map was constructed using Cytoscape 3.7.1 (www.cytoscape.org) [105]. The Smp identifiers for all *S*. *mansoni* phosphoproteins were imported into Cytoscape and potential interactions retrieved using the StringApp plug-in with a confidence threshold >0.7 [44]. The Clusterviz MCODE clustering algorithm was then applied to the retrieved data to visualize highly interconnected regions within the network using default parameters. These regions, representing sub-networks were then interrogated for functional annotation using the Cytoscape app “STRING enrichment” with redundant annotations removed. Networks for selected *S*. *mansoni* protein kinases were then imported into Cytoscape using StringApp (confidence 0.7; maximum 50 additional interactors) and networks merged with the global phosphoproteome to identify phosphoproteins within the kinase network.

### Western blotting

A lithium dodecyl sulfate (LDS) sample buffer (5x) (Invitrogen) was mixed with the adult worm homogenate and samples were heated to 95°C for 5 min, sonicated for 30 s, and equal amounts of protein (15 μg) electrophoresed on 10% Precise sodium dodecyl sulfate polyacrylamide electrophoresis gels (Invitrogen). Proteins were transferred to nitrocellulose membranes, blocked in 5% non-fat dried milk, washed in Tween-Tris-buffered saline (TTBS) and incubated in overnight at 4°C in phospho-PKA substrate (#9624, CST), phospho-PKC substrate (#2261, CST), or phospho-Akt substrate (#9614, CST) motif antibodies. Blots were then washed in TTBS, incubated in horseradish peroxidase (HRP)-conjugated secondary antibodies (CST; 1:3000; 2 h), and visualized using West Pico (Thermo Scientific) substrate on a GeneGnome (Syngene) chemiluminescence imaging system.

### Production of CelluSpots custom peptide array for *S*. *mansoni*

The aim was to generate a signalling-biased peptide array for screening *S*. *mansoni* protein kinase activities. The phosphopeptide data set was submitted to Scansite (www.scansite4.mit.edu) to identify potential upstream protein kinases responsible for the identified phosphorylation events. The Scansite-predicted phosphorylation site within each peptide was then screened against the original phosphopeptide data to ensure correct phosphosite match; peptides with mismatched sites were excluded. Results were then filtered for interesting protein kinases to be represented on the array. Each 15 mer peptide selected for the array was then resubmitted to Scansite, interrogating for mammalian and/or yeast upstream kinases. Sometimes this revealed a possible additional upstream protein kinase for a particular peptide. Furthermore, for peptides possessing more than one predicted phosphorylation site, the additional site was considered valid only when the site was also phosphorylated in the experimental data.

To identify further peptides for inclusion on the array, the experimental data tables were manually screened to identify additional peptides within signalling-related proteins. The full protein sequence for each (Smp) candidate was retrieved and submitted to Phospho.ELM via PhosphoBLAST [106], and also to Scansite, to identify putative upstream kinases. In some cases, no upstream kinase for a phosphosite was predicted; nevertheless, if the protein was deemed interesting in terms of schistosome biology (e.g. Smp_105410—SGTP4 [15]), the relevant phosphopeptide was flagged for inclusion on the array. Finally, additional peptide substrates were included based on our knowledge of schistosome protein kinase substrate preferences ([14,15,47]). All protein kinase names on the array were checked/updated according to the terminology used in the *S*. *mansoni* kinome paper [18]. Finally, gene expression data for each protein represented on the array was obtained from GeneDB (www.genedb.org). The final *S*. *mansoni* custom “CelluSpots” 96-spot peptide array was printed under contract by Intavis Bioanalytical Instruments AG. Each slide comprises two sub-arrays, each with identical peptides.

### Profiling of *S*. *mansoni* kinase activities

Separated adult male and female *S*. *mansoni* were homogenized on ice in 1 x cell lysis buffer (CST; 10 μl/worm) containing HALT protease and phosphatase inhibitor cocktails (EDTA-free; ThermoFisher Scientific) using a motorized microfuge pestle. Cooled homogenates were then centrifuged at 14,000 x *g* for 3 min and the supernatants recovered. An aliquot was removed for protein estimation using the detergent compatible Bradford Assay (ThermoFisher Scientific) and samples were stored at -80°C.

Peptide arraying was conducted as detailed elsewhere [58,107] with modifications. The arrays were blocked with 10% BSA in TTBS overnight at 4°C on a rocker. They were then washed twice, 5 min each with agitation, in array wash buffer (1 M NaOAC, 1% BSA, in TTBS). The protein concentrations of each parasite sample were next equalized using cell lysis buffer and the samples were prepared for arraying by combining ten or five male worm protein equivalents (typically ~50 μl or 25 μl, respectively at ~2 μg/μl) with 1 x kinase buffer (CST) and 2 μl of 10 mM ATP (CST) to provide a final volume of 200 μl. The array was removed from wash buffer, drained onto tissue and each sample gently pipetted onto the array. A LifterSlip (25 mm x 75 mm; Electron Microscopy Sciences) was applied on top of the sample and arrays were incubated in a humidified box at 37°C with gentle shaking for 3 h. Thereafter, arrays were washed in array wash buffer twice for 5 min each.

To enable the detection of phosphopeptides, the arrays were incubated in a solution containing Phos-tag biotin complexed to streptavidin HRP. 10 μl Phos-tag Biotin BTL-111 (1 mM aqueous solution; Wako Chemicals) was combined with 20 μl 1 mM ZnCl_2_ (Sigma), 1 μl HRP-streptavidin (GE Healthcare) and 469 μl TTBS and incubated at room temperature for 30 min. The solution was then subject to ultrafiltration through a NanoSep 30K Omega centrifugal filter (14,000 x *g*, 20 min; Pall Corporation) and the recovered Phos-tag biotin-streptavidin HRP complex diluted with 15 ml array wash buffer and stored at 4°C. Arrays were removed from wash buffer, drained, and incubated in the detection solution for 1 h at room temperature on a rocker before further washing (twice, 5 min each) in array wash buffer. Phosphorylated peptides on the array were then detected using ECL Prime (GE Healthcare) and a GBox imaging system (chemiluminescence mode; Syngene) and images captured. The intensity of each peptide spot on the array was quantified using the ImageJ plugin “Protein Array Analyzer” (http://image.bio.methods.free.fr/ImageJ/?Protein-Array-Analyzer-for-ImageJ), using default parameters and auto spot detection. The sub-arrays were analysed independently from one another and spot intensities within each array/sub-array were normalized to the darkest spot, which was assigned a value of 1. Arrays from two independent experiments were analysed and statistical comparisons were done using student’s t-test.

## Declarations

### Data availability statement

Data generated or analysed during the current study are included in this published article [and its supplementary information files].

## Supporting information

S1 Fig Suitability of *S*. *mansoni* protein extracts for phosphoproteomic profiling. Protein extracts from the three separate batches (lanes in each panel left to right; 15 μg total protein in each lane) of adult *S*. *mansoni* were prepared and processed for western blotting with: (A) Phospho-PKA substrate, (B) phospho-PKC substrate, and (C) phospho-Akt substrate antibodies to confirm that proteins derived from each separate worm batch were of sufficient quality for phosphoproteomic analysis.
(PDF)

S2 Fig Mapping of phosphorylated proteins to KEGG pathways in Blast2GO reveals enrichment in numerous biosynthetic, metabolic and signalling pathways. The top 30 pathways (based on number of sequences) are shown.
(PDF)

S3 Fig Molecular complex detection (MCODE) of the *S*. *mansoni* phosphoprotein interactome. ClusterViz revealed highly interconnected sub-networks that were then functionally annotated using STRING enrichment; clusters 5–10 are displayed (clusters 1–4 and the complete interactome can be visualized in Fig 7). Examples of the highest scoring KEGG pathway matches for each MCODE cluster are listed.
(PDF)

S1 Table LC/MS-MS data for phosphotyrosine IAP enrichment. Includes i) Column Definitions tab, ii) a redundant list of all MS/MS identifications with accompanying LC-MS/MS acquisition data and peptide assignment scoring data (Details tab), and iii) a tab non-redundant by protein/site (Summary tab).
(XLSX)

S2 Table LC/MS-MS data for IMAC-Fe^3+^ enrichment. Includes i) Column Definitions tab, ii) a redundant list of all MS/MS identifications with accompanying LC-MS/MS acquisition data and peptide assignment scoring data (Details tab), and iii) a tab non-redundant by protein/site (Summary tab).
(XLSX)

S3 Table LC/MS-MS data for phosphothreonine/phosphoserine IAP enrichment (performed with phosphotyrosine IAP flow-through). Includes i) Column Definitions tab, ii) a redundant list of all MS/MS identifications with accompanying LC-MS/MS acquisition data and peptide assignment scoring data (Details tab), and iii) a tab non-redundant by protein/site (Summary tab).
(XLSX)

S4 Table Sequence motifs and putative upstream protein kinases. Significantly enriched phosphorylation motifs were generated using the Motif-X algorithm using the *S*. *mansoni* full proteome as background. Tabs are provided for Y, T, S centered motifs for both the standard and degenerate amino acid set. Upstream kinases for known motifs were extracted using the HPRD motif finder and Phosida.
(XLSX)

S5 Table *S*. *mansoni* protein kinases containing one or more phosphorylated residue. Annotation of kinase (protein description/group/family) to Smp_ identifier was based on Andrade et al [18] and Grevelding [20]. The phosphorylation sites were extracted from the raw data tables (S1–S3 Tables) and the relative positions of activation loops within each protein identified using NCBI BLASTp (conserved protein domain tool) and InterPro; phosphorylated residues within the activation loops are highlighted in red.
(XLSX)

S6 Table Distribution of the *S*. *mansoni* phosphoproteins in KEGG pathway database, derived using Blast2GO. (XLSX)

S7 Table Details of interacting phosphoproteins (nodes) derived from Cytoscape (0.7 confidence threshold). (XLSX)

S8 Table Data tables for molecular complex detection (MCODE) using ClusterViz (Cluster tab) and STRING enrichment data (Enriched Proteins in Clusters tab). (XLSX)

S9 Table Interacting proteins for selected kinases (EGFR, PKCβ, ERK2, Akt, and PKA), using a maximum 50 interactors for each query (seed) protein and high confidence (>0.7) interaction score. Proteins (nodes) coloured red represent those phosphorylated amongst the total interaction network, with the seed node coloured yellow. Detailed annotation of protein kinases (corresponding to Smp_ identifier) was based on Andrade et al [18] and Grevelding [20].
(XLSX)

S10 Table Selected peptides for inclusion on the Celluspots peptide array. The +7/-7 peptide sequence is shown together with the substrate name, its Smp identifier, phosphorylation site, and the Table from which the peptide was derived (Tables 1–3 refer to S1–S3 Tables, respectively). The upstream protein kinase for each substrate is given where a prediction exists (predicted kinase) based on Scansite, Phospho.ELM analysis, or otherwise. Peptides 71–93 lack putative upstream protein kinase annotation.
(XLSX)

S11 Table Layout and content of the custom Celluspots peptide array containing peptide substrates specific to *S*. *mansoni*. Each slide comprises two sub-arrays each with identical peptides (A1-A24, B1-B24, C1-C24, D1-D24). Includes i) a Peptides tab that lists the peptides and the proteins from which they are derived, and ii) Chip 2x96 tab, which overviews the array layout.
(XLS)
