# Supplementary figures and images for: Deep phosphoproteome analysis of Schistosoma mansoni leads development of a kinomic array that highlights sex-biased differences in adult worm protein phosphorylation
Source: PLoS Negl Trop Dis. 2020 Mar 23;14(3):e0008115. doi: 10.1371/journal.pntd.0008115 (PMC7089424; doi:10.1371/journal.pntd.0008115)

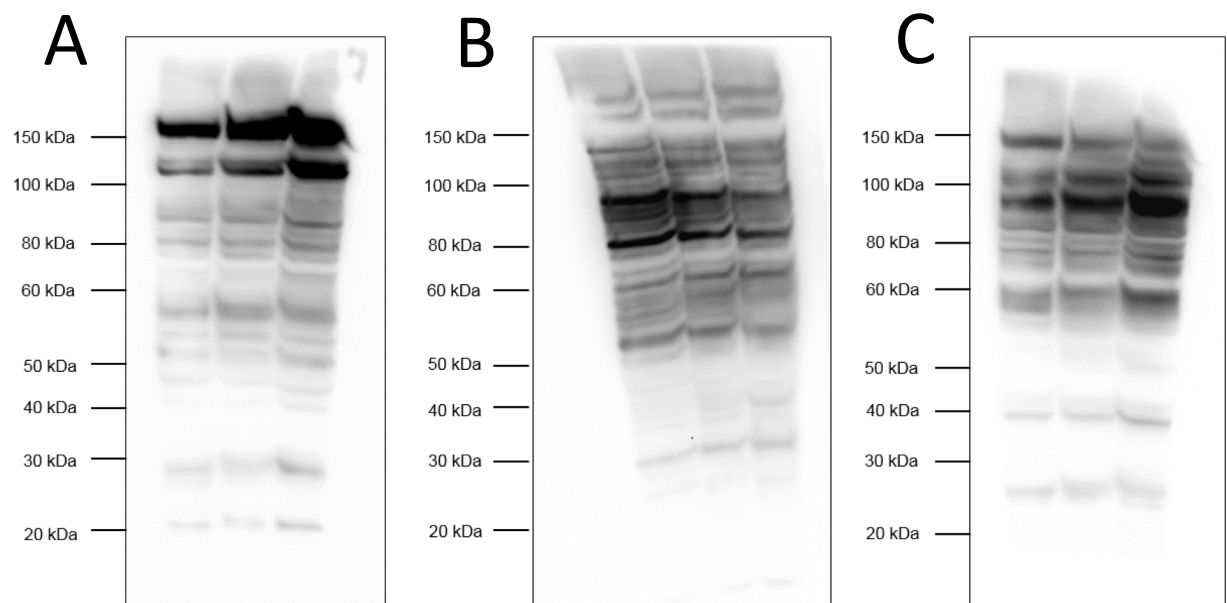

Supplement: S1 Fig — Protein extracts from the three separate batches (lanes in each panel left to right; 15 μg total protein in each lane) of adult S. mansoni were prepared and processed for western blotting with: (A) Phospho-PKA substrate, (B) phospho-PKC substrate, and (C) phospho-Akt substrate antibodies to confirm that proteins derived from each separate worm batch were of sufficient quality for phosphoproteomic analysis. (PDF) [file pntd.0008115.s001.pdf]

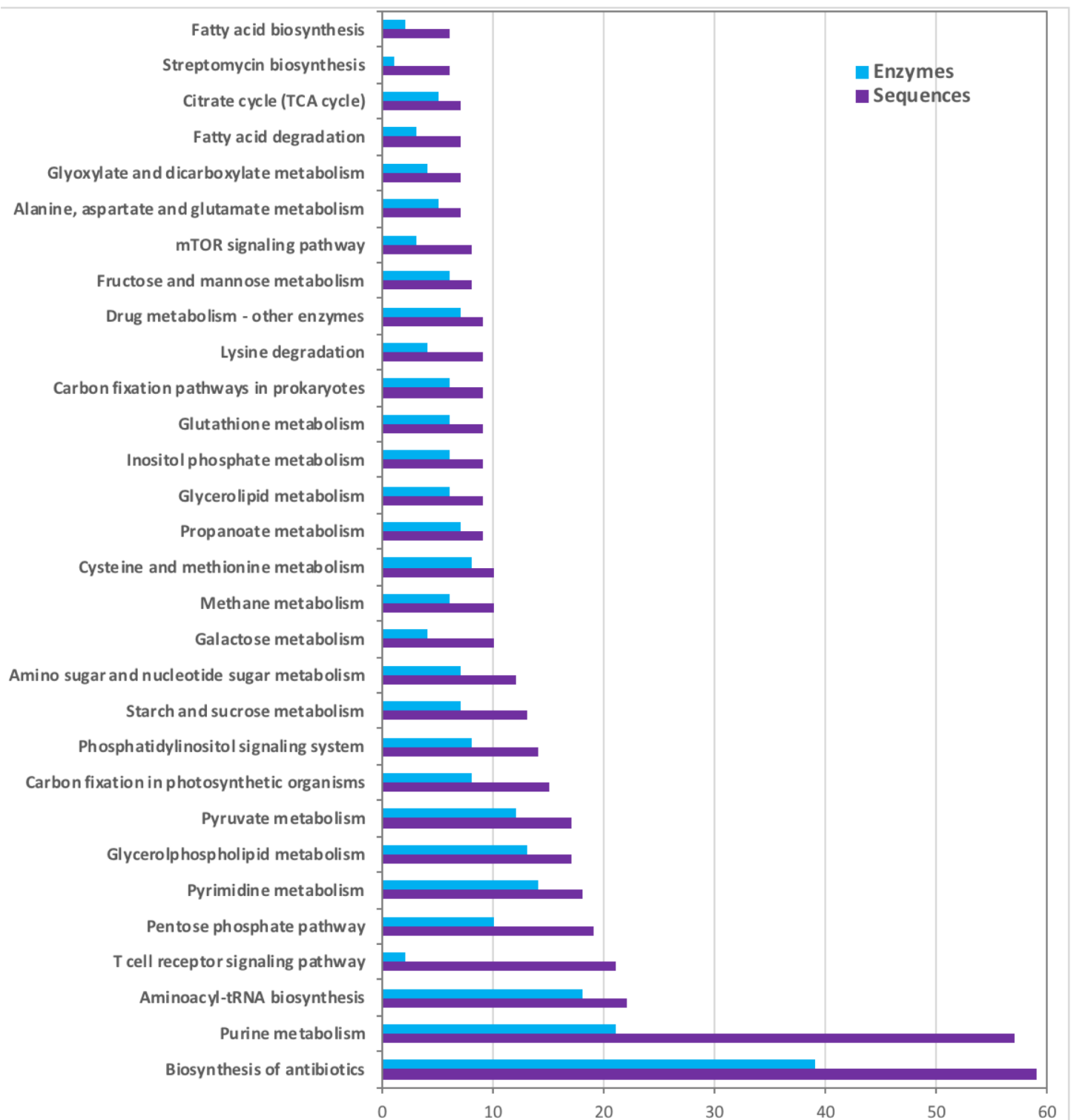

Supplement: S2 Fig — The top 30 pathways (based on number of sequences) are shown. (PDF) [file pntd.0008115.s002.pdf]
