# Supplementary material for: Deep phosphoproteome analysis of Schistosoma mansoni leads development of a kinomic array that highlights sex-biased differences in adult worm protein phosphorylation
Source: PLoS Negl Trop Dis. 2020 Mar 23;14(3):e0008115. doi: 10.1371/journal.pntd.0008115 (PMC7089424; doi:10.1371/journal.pntd.0008115)
Supplement: S3 Fig — ClusterViz revealed highly interconnected sub-networks that were then functionally annotated using STRING enrichment; clusters 5–10 are displayed (clusters 1–4 and the complete interactome can be visualized in Fig 7). Examples of the highest scoring KEGG pathway matches for each MCODE cluster are listed. (PDF) [file pntd.0008115.s003.pdf]

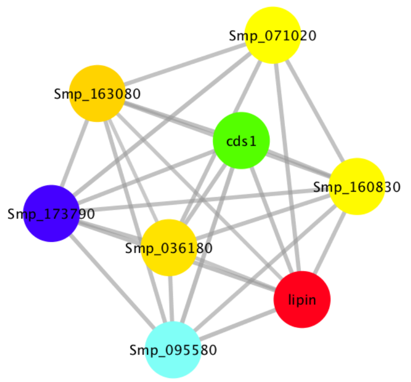

MCODE cluster 5
Nodes: 8; edges: 26; score ~7.4
KEGG: Glycerophospholipid metabolism (8/8); phosphatidylinositol signaling (3/8)

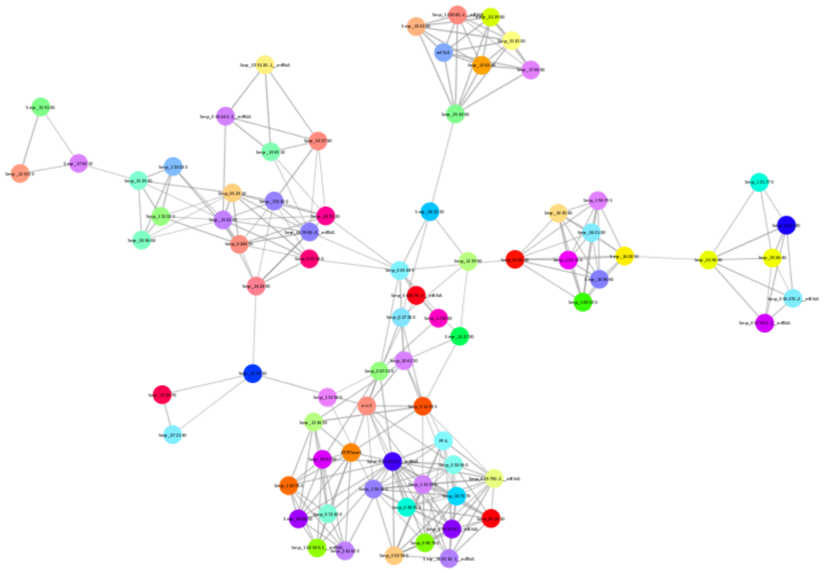

MCODE cluster 6
Nodes: 77; edges: 268; score ~7.1
KEGG: RNA transport (15/77); ubiquitin mediated proteolysis (10/77); purine metabolism (10/77) glycolysis (7/77)

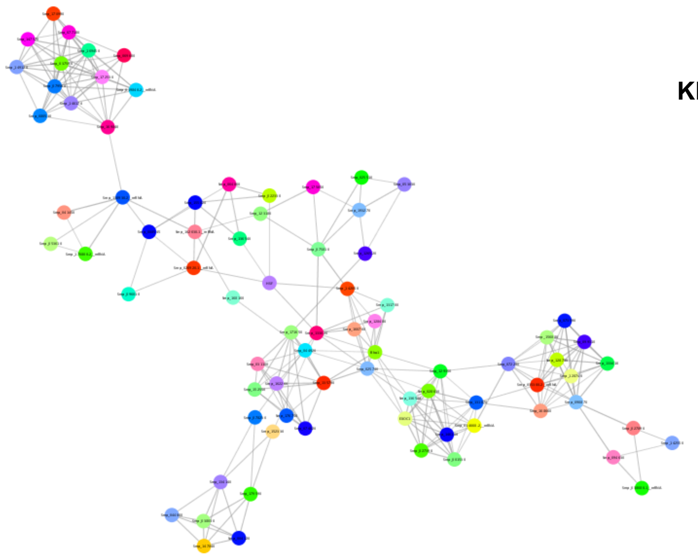

MCODE cluster 7
Nodes: 80; edges: 258; score ~6.5
KEGG: Endocytosis (7/80); DNA replication (5/80)

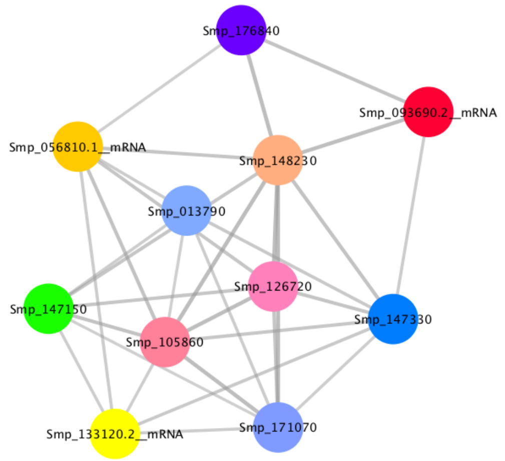

MCODE cluster 8
Nodes: 11; edges: 32; score ~6.4
KEGG: Spliceosome (6/8)

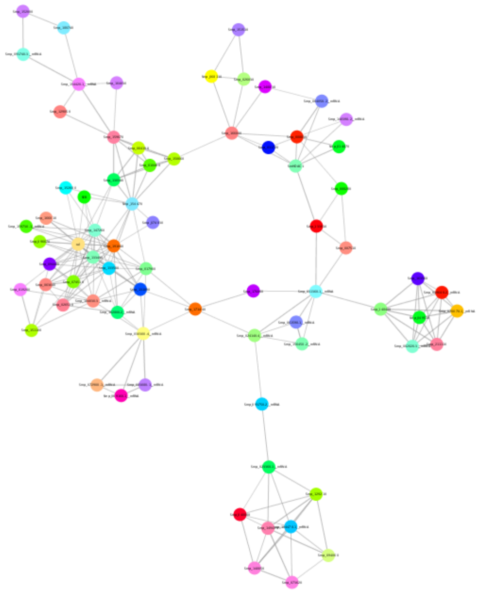

MCODE cluster 9
Nodes: 73; edges: 204; score ~5.7
KEGG: mRNA surveillance (8/73); inositol phosphate metabolism (6/73); aminoacyl-tRNA biosynthesis (6/73)

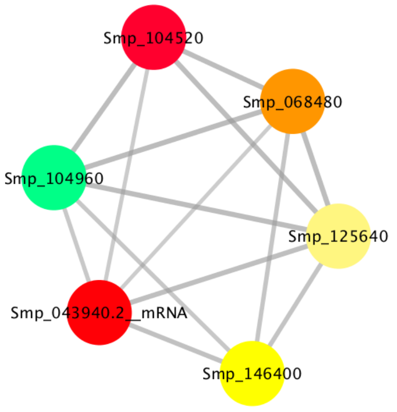

MCODE cluster 10
Nodes: 6; edges: 14; score ~5.6
KEGG: SNARE interactions in vesicular transport (3/6)
